# A pH-responsive GelMA-KerMA hydrogel delivering luteolin-loaded PLGA nanoparticles promotes myocardial repair via Akt/Bad signaling and multitarget regulation

**DOI:** 10.1016/j.mtbio.2026.103791

**Published:** 2026-10-31

**Authors:** Dongbin Jiang, Jiaying Li, Junkai Hu, Dan Jiang, Weihu Yang, Wenhua Huang

**Affiliations:** a Department of Surgery, The First School of Clinical Medicine, Guangdong Medical University, Zhanjiang, China; b Guangdong Engineering Research Center for Translation of Medical 3D Printing Application, Guangdong Provincial Key Laboratory of Digital Medicine and Biomechanics, National Key Discipline of Human Anatomy, School of Basic Medical Sciences, Southern Medical University, Guangzhou, China; c Key Laboratory of Biorheological Science and Technology, Ministry of Education, College of Bioengineering, Chongqing University, Chongqing, China

**Keywords:** pH-responsive hydrogel, PLGA nanoparticles, Luteolin, Akt signaling cascade, Myocardial infarction, Multifunctional drug delivery

## Abstract

Myocardial infarction (MI) triggers cardiomyocyte apoptosis, oxidative stress imbalance, ventricular fibrosis, and impaired angiogenesis. Luteolin, a natural flavonoid compound, possesses antioxidant, anti-apoptotic, and cardioprotective properties, making it a promising candidate for MI treatment. However, its poor water solubility, low bioavailability, and chemical instability severely hinder its clinical translation. To address these limitations, we developed a photo-crosslinked composite hydrogel delivery system using gelatin methacryloyl and keratin methacryloyl (GelMA-KerMA). Luteolin-loaded poly(lactic-co-glycolic acid) (PLGA) nanoparticles were uniformly dispersed within a three-dimensional gel network to achieve pH-triggered local drug delivery. The covalent GelMA–KerMA network retained structural integrity under both pH conditions. In the weakly acidic infarct microenvironment, acid-catalyzed hydrolysis of PLGA ester bonds was the primary driver of accelerated luteolin release, whereas the network mainly acted as a sustained-retarding reservoir. The incorporation of KerMA endowed the patch with enhanced mechanical stability and cytocompatibility, enabling better mechanical matching with the infarcted myocardium. The composite hydrogel promotes myocardial repair and functional recovery through the coordinated regulation of the rapid Akt/Bad phosphorylation cascade. It also sustains MAPK/Rap1-dependent transcriptional reprogramming. It inhibits mitochondrial apoptosis, scavenges reactive oxygen species (ROS), suppresses fibroblast activation, and promotes neovascularization. This study presents a protein-based delivery platform that integrates tailored physicochemical and mechanical properties with multi-pathway myocardial repair capabilities, offering a biomaterial strategy for minimally invasive local drug delivery in ischemic MI.

## Highlights

A dual-network GelMA-KerMA hydrogel adjusts swelling through carboxyl protonation, while PLGA acid hydrolysis drives pH-accelerated luteolin release.

Luteolin-loaded PLGA nanoparticles improve aqueous dispersion and bioavailability and enable multi-target cardioprotection.

The hydrogel inhibits mitochondrial apoptosis via Akt/Bad, suppresses fibrosis, and promotes angiogenesis, improving cardiac function in a rat MI model.

## Graphical abstract

**Figure:**
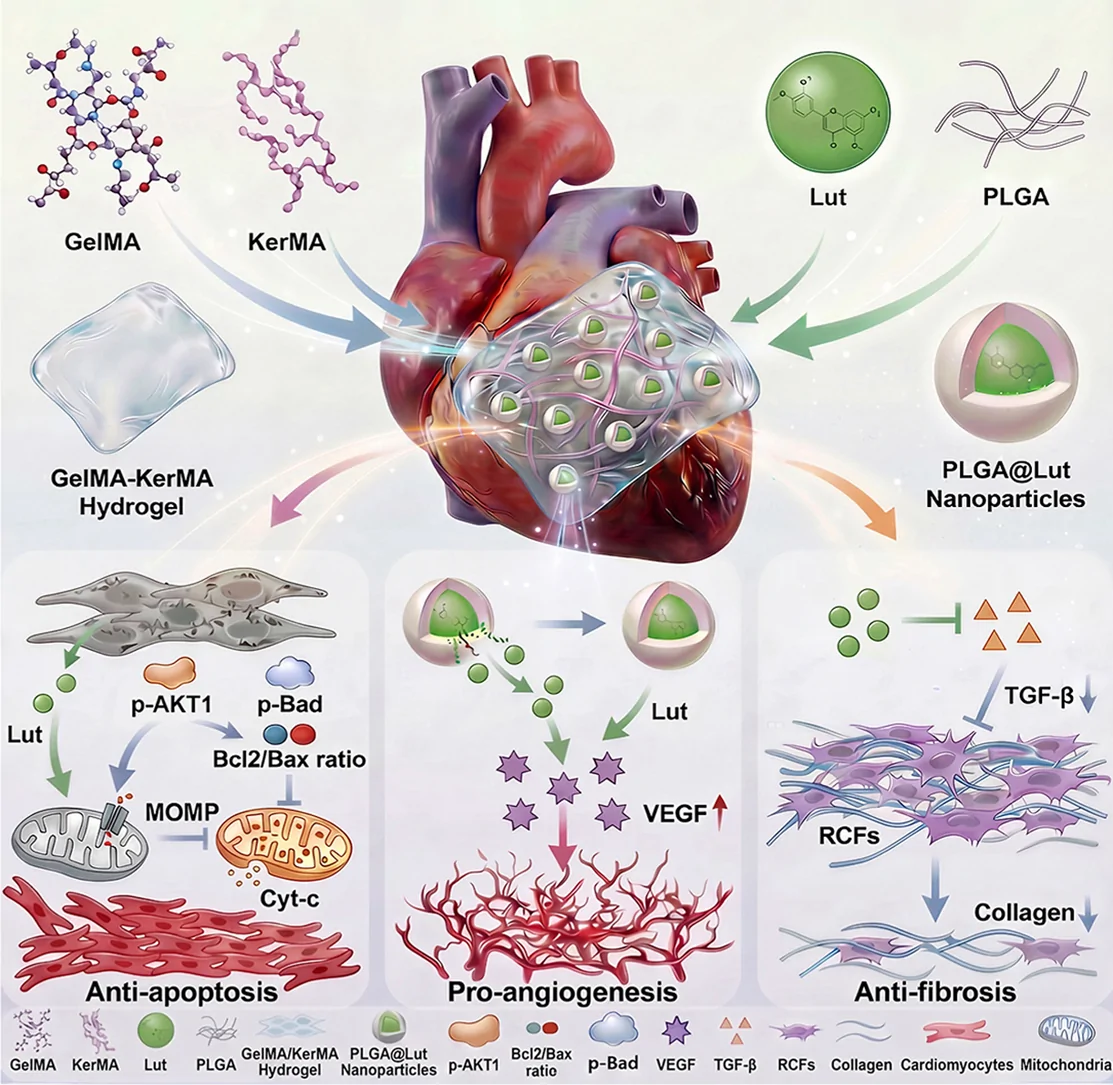

## 1 Introduction

Interruption of blood perfusion caused by acute coronary artery occlusion induces severe myocardial ischemia and hypoxia. This leads to irreversible cardiomyocyte death, intense oxidative stress, insufficient vascular repair, and adverse ventricular remodeling [1], [2]. Although coronary intervention can partially restore circulation, such approaches remain unable to reverse ongoing cardiomyocyte loss and irreversible fibrotic scar formation. Ultimately, this results in deteriorating cardiac function and poor long-term prognosis [3]. Over the past several decades, pharmacological agents such as β-blockers and angiotensin-converting enzyme inhibitors have been used to ameliorate pathological remodeling after myocardial infarction (MI). However, they fail to significantly improve outcomes [4]. Cardiac transplantation represents the only viable surgical option, but its clinical application is severely limited by postoperative immune rejection and donor scarcity [5]. Therefore, identifying new strategies to inhibit ventricular remodeling after MI is of paramount importance.

Cardiac patches provide mechanical support to the infarcted wall, suppress pathological remodeling, and enable local delivery of cells, drugs, or exosomes, thereby enriching therapeutic agents at the lesion while minimizing systemic exposure [6], [7]. Gelatin methacryloyl (GelMA) offers good cell adhesion and tissue-matching properties [8], and compounding it with functional nanomaterials can further improve hydrogel performance [9], [10], [11]. However, most existing patches are passive release systems and cannot respond intelligently to the infarct microenvironment [12]; lesion-responsive patches remain an urgent developmental direction.

Accumulating evidence indicates that luteolin, through its multitarget properties, can simultaneously intervene in multiple pathological pathways of MI, thereby exerting cardioprotective effects [13], [14]. However, free luteolin suffers from poor water solubility and low bioavailability, which severely limits its local administration at cardiac injury sites. Nanoparticle-based delivery systems offer a promising strategy for significantly enhancing therapeutic efficacy [15]. Poly(lactic-co-glycolic acid) nanoparticles (PLGA NPs) are widely used as drug carriers owing to their excellent biodegradability and tunable drug release kinetics.

After acute MI, interruption of coronary perfusion forces ischemic cardiomyocytes toward anaerobic glycolysis. This generates lactate and protons that cannot be removed because perfusion-dependent washout is impaired. Ongoing inflammatory-cell activation and metabolic CO_2_ production further accumulate extracellular H^+^ and lower interstitial pH. This post-ischemic acidosis begins within minutes of occlusion, deepens through the acute phase over hours to days, and persists at approximately pH 6.5–6.8 over days to weeks during the remodeling phase, producing a lesion-restricted, temporally sustained acidic window rather than a transient instantaneous drop [16], [17]. This pathophysiological pH trajectory provides the chemical basis for a lesion-responsive delivery design and motivated the use of pH 6.5 as the representative ischemic condition in vitro. Liu et al. [18] developed a pH-responsive hydrogel based on amide crosslinking loaded with platelet-rich plasma (PRP) to alleviate ventricular remodeling after MI. Cheng et al. [19] reported a pH-responsive conductive hydrogel delivering metformin and exosomes to enhance cardiac repair after myocardial ischemia-reperfusion injury. pH-responsive hydrogels based on dynamic covalent bonds have attracted attention for their reversible cleavage under acidic conditions [20], but these materials face complex synthesis, limited long-term stability, and potential toxicity [21]. In contrast, pH-responsive systems based on physical interactions (e.g., hydrogen bonding) offer facile synthesis and high biocompatibility [22].

To address these limitations, we developed a relatively simple pH-responsive strategy: a GelMA-keratin methacryloyl (KerMA) composite hydrogel loaded with luteolin-loaded PLGA nanoparticles (GK@NPs) for the localized treatment of MI. GelMA was crosslinked with KerMA to fabricate a scaffold with good mechanical properties and biocompatibility. The composite hydrogel forms a permanent covalent backbone via photo-crosslinking, supplemented by a secondary hydrogen-bonded network. The pH dependence of GelMA and KerMA involves two distinct levels: (1) at the network level, reversible protonation/deprotonation of abundant carboxyl groups on GelMA/KerMA chains modulates electrostatic repulsion, mesh size, and swelling/compliance but does not cleave the permanent covalent backbone; (2) at the nanoparticle level, acid-catalyzed hydrolysis of PLGA ester bonds drives irreversible luteolin (Lut) release and particle erosion, and the resulting local voids facilitate drug diffusion and gradual surface erosion. This two-level design avoids the synthetic complexity and potential toxicity of dynamic-covalent systems and enables pH-triggered multistage release.

## 2 Materials and methods

### 2.1 Materials and reagents

PLGA (LA: GA = 50:50, weight-average molecular weight Mw ≈ 20-40 kDa) was purchased from Jinan Daigang Bioengineering Co., Ltd. (Shandong, China). Luteolin (purity > 98%) was purchased from Shanghai Tauto Biotech Co., Ltd. (Shanghai, China). Gelatin (derived from porcine skin, Type A), methacrylic anhydride (MA), lithium phenyl-2,4,6-trimethylbenzoylphosphinate (LAP), dichloromethane, and polyvinyl alcohol were all analytical grade. They were purchased from Beijing Bailingwei Technology Co., Ltd. (Beijing, China) and Shanghai Aladdin Biochemical Technology Co., Ltd. (Shanghai, China), respectively. Keratin (from wool) was purchased from Tokyo Chemical Industry Co., Ltd. (TCI, Tokyo, Japan). DiR (near-infrared fluorescent probe for cell membrane) was purchased from UElandy (Guangzhou, China). MK-2206 dihydrochloride (Akt1/2/3 inhibitor) was purchased from Selleck Chemicals (Houston, USA). All other routine chemical reagents not specifically noted were purchased from Sigma-Aldrich (Shanghai, China). Cell culture media (Dulbecco's modified Eagle's medium (DMEM), high glucose, F-12 Nutrient Mixture), fetal bovine serum (FBS), and trypsin (Trypsin-EDTA) were purchased from Thermo Fisher Scientific (China). Cell Counting Kit-8 (CCK-8), reactive oxygen species (ROS) assay kit, Annexin V-fluorescein isothiocyanate (FITC)/propidium iodide (PI) apoptosis detection kit, JC-1 mitochondrial membrane potential assay kit, 5-ethynyl-2′-deoxyuridine (EdU) cell proliferation assay kit, and Calcein-AM/PI live/dead cell staining kit were all purchased from Beyotime Biotechnology (Shanghai, China). Primary antibodies and corresponding secondary antibodies for Western blotting and immunofluorescence staining were purchased from Proteintech Group (Wuhan, China) and Shenyang Wanlei Biotechnology Co., Ltd. (Liaoning, China), respectively. The one-step terminal deoxynucleotidyl transferase dUTP nick-end labeling (TUNEL) cell apoptosis detection kit (CF488 green fluorescence) was purchased from Wuhan Servicebio Technology Co., Ltd. (Wuhan, China). Human umbilical vein endothelial cells (HUVECs), rat cardiomyoblast cell line H9C2, and rat cardiac fibroblasts (RCFs) were purchased from Wuhan Procell Life Science & Technology Co., Ltd. (Procell, Wuhan, China).

### 2.2 Preparation and characterization of Lut-PLGA NPs

Lut-PLGA NPs were prepared by the oil-in-water (O/W) emulsion-solvent evaporation method. PLGA (100 mg) and luteolin (10 mg) were dissolved in dichloromethane and emulsified in 1% (w/v) PVA by high-speed shear (15,000 rpm, 2–3 min, ice bath) followed by probe sonication (200 W, pulse mode, 2 min). After centrifugation (12,000 rpm, 20 min, 4°C), particles were washed three times with deionized water and lyophilized. Chemical structure was examined by FTIR; hydrodynamic diameter, polydispersity index, and zeta potential were measured by DLS and electrophoretic light scattering (Malvern Zetasizer Nano ZS90); morphology was observed by negative-staining TEM (JEM-2100Plus).

### 2.3 Determination of encapsulation efficiency and drug loading

The UV-Vis spectrum of luteolin showed a characteristic absorption peak at 355 nm. A calibration curve was constructed in PBS (pH 7.4, containing 5% DMSO): A = 0.128C + 0.0002 (R^2^ = 0.998). Free luteolin was separated by ultrafiltration (MWCO 10 kDa, 20,000 × g, 30 min, 4°C), and total luteolin was extracted with acetonitrile-DMSO (1:1, v/v) under sonication. Encapsulation efficiency (EE) and drug loading (DL) were calculated as follows:

EE% = (W_total − W_free) / W_total × 100%

DL% = (W_total − W_free) / W_nanoparticles × 100%

### 2.4 Synthesis of GelMA

GelMA was synthesized as reported [23]: 10% (w/v) Type A gelatin in 0.25 M carbonate-bicarbonate buffer (pH 9.4) reacted with 8 mL methacrylic anhydride (0.5 mL/min, 50°C, 3 h), then was dialyzed (MWCO 12–14 kDa) against deionized water for 5 days and lyophilized; methacryloyl substitution was ≈73% by ^1^H NMR [24].

### 2.5 Synthesis of KerMA

KerMA was methacryloylated analogously to GelMA [25]: 2% (w/v) wool-derived soluble keratin in 0.25 M carbonate-bicarbonate buffer (pH 9.4) reacted with methacrylic anhydride (0.6 mL/g keratin, 45°C, 3 h, protected from light), then was diluted, dialyzed, and lyophilized; methacryloyl substitution was ≈80% by ^1^H NMR [25].

### 2.6 Preparation of GK composite hydrogels

GK composite hydrogels were prepared using a one-step blending and photo-crosslinking method. GelMA and KerMA powders were co-dissolved in sterile PBS (pH 7.4) at 40°C under continuous stirring until complete dissolution, yielding three precursor solutions of different compositions. In all solutions, the final concentration of GelMA was fixed at 5% (w/v), while the final concentrations of KerMA were 0.5%, 1.0%, and 1.5% (w/v), respectively. LAP was then added to each solution to a final concentration of 0.25% (w/v) and mixed thoroughly by vortexing. Photo-crosslinking was achieved by UV irradiation for 60 s. The resulting hydrogels were designated as GK-0.5, GK-1.0, and GK-1.5 according to KerMA concentration.

### 2.7 Preparation of pH-Responsive GK@NPs composite hydrogel

The drug-loaded composite hydrogel was prepared based on the formulation described in Section 2.6. Pre-prepared and lyophilized Lut-PLGA NPs powder was added to the GK-0.5 precursor solution at a final concentration of 500 μg/mL. Under 40°C, light-protected conditions, the mixture was magnetically stirred at 200 rpm and photo-crosslinked under the same conditions as described in Section 2.6 to obtain the drug-loaded composite hydrogel (GK@NPs).

### 2.8 Physicochemical characterization of composite hydrogels

The rheological properties of the composite hydrogels were measured at 37°C using a rotational rheometer (MCR302, Anton Paar, Austria). Amplitude sweeps (strain range: 0.1%-100%, fixed frequency: 1 Hz) were performed to identify the linear viscoelastic region (LVR), followed by frequency sweeps (frequency range: 0.1-10 Hz, fixed strain: 1%) within the LVR. Additionally, steady-state shear tests (shear rate: 0.1-100 s^−1^) were conducted to evaluate shear-thinning behavior. To investigate the pH responsiveness of the composite hydrogels, all rheological tests were performed in PBS at both pH 6.5 and pH 7.4.

The microscopic morphology and elemental distribution of the hydrogels were observed and analyzed using scanning electron microscopy (SEM, Sigma 360, ZEISS, Germany) equipped with an energy-dispersive X-ray spectroscopy (EDS) detector. The samples were lyophilized, sputter-coated with gold, imaged at designated accelerating voltages, and line-scanned by EDS to analyze the spatial distribution of specific elements. X-ray photoelectron spectroscopy (XPS, ESCALAB 250Xi, Thermo Fisher Scientific, USA) was employed for qualitative and semi-quantitative analysis of the elemental composition and chemical valence states of the hydrogel surfaces. All spectra were charge-corrected to the C 1s binding energy (284.8 eV).

### 2.9 Cell spreading behavior analysis

Precursor solutions of different composite hydrogel compositions were directly added to the bottom of confocal dishes and UV-crosslinked for 60 s. The crosslinked hydrogels were equilibrated in complete culture medium at 37°C for 12 h to stabilize their surface properties. H9C2 cells and RCFs were then seeded on the surface of each hydrogel group at a density of 1 × 10^4^ cells/cm^2^ and cultured for 24 h at 37°C with 5% CO_2_. After culture, the medium was discarded, and cells were gently washed with PBS, fixed with 4% paraformaldehyde (PFA) at room temperature (RT) for 15 min, and permeabilized with 0.1% Triton X-100 at RT for 5 min. All nuclei were labeled with 4′,6-diamidino-2-phenylindole (DAPI), and the actin cytoskeleton (F-actin) was labeled with phalloidin. To specifically label RCFs, anti-Vimentin antibody was additionally used. After staining, cells were washed with PBS and imaged using confocal microscopy. Images were analyzed using ImageJ software. To accurately assess the single-cell spreading area, only clearly defined isolated single cells were selected for measurement, excluding cell clusters in contact with one another, and their projected areas were calculated.

### 2.10 Biocompatibility of composite hydrogels

Sterilized hydrogels of different compositions were incubated in cell culture medium for 24 h. H9C2 cells and RCFs were pre-seeded in 96-well plates and allowed to adhere for 24 h. Following pre-incubation, extracts from GelMA, GK, GK@PLGA, and GK@NPs hydrogels were added to respective wells. After 1 day, cell viability was assessed by CCK-8 assay via absorbance measurement at 450 nm using a microplate reader. For direct-contact cytotoxicity assays, cells were stained according to the Calcein-AM/PI dual-staining kit protocol for 30 min. Calcein-AM labeled live cells (green fluorescence) and PI labeled dead cells (red fluorescence).

Fresh blood was collected from healthy rats to prepare a 1.0% (w/v) erythrocyte suspension. A volume of 500 μL of the erythrocyte suspension was added to an equal volume of each hydrogel extract. The negative control was an equal volume of PBS, and the positive control was an equal volume of deionized water. All samples were incubated at 37°C for 1 h, then centrifuged, and the absorbance of the supernatant was measured at 545 nm using a microplate reader. The hemolysis ratio was calculated as: hemolysis ratio (%) = [(OD_sample - OD_negative)/(OD_positive - OD_negative)] × 100%.

### 2.11 Swelling and degradation behavior of composite hydrogels

Hydrogel samples of each composition were lyophilized, and their initial dry mass (W_d) was recorded. Samples were fully immersed in 5 mL of PBS at the corresponding pH and placed on a constant-temperature shaker at 37°C. At predetermined time points, the samples were removed, excess surface water was gently blotted with filter paper, and the swollen mass (W_t) was immediately recorded. The swelling ratio (SR) was calculated as follows: SR (%) = [(W_t - W_d)/W_d] × 100%.

Degradation was assessed for both the GK-0.5 matrix and the GK@NPs composite at pH 7.4 and pH 6.5. For the degradation studies, the initial dry mass (W_0) of the hydrogel samples was recorded before immersion in 5 mL of PBS at the corresponding pH on a 37°C shaker. At regular intervals, the samples were removed, gently rinsed with deionized water to remove soluble degradation products, lyophilized, and the current dry mass (W_t) was recorded. PBS was completely replaced at regular intervals throughout the degradation period to maintain a constant pH and sink conditions. The cumulative degradation rate (CDR) was calculated as follows: CDR (%) = [(W_0 − W_t)/W_0] × 100%, representing the percentage of initial dry mass lost through degradation up to time t.

### 2.12 In vitro drug release profile of composite hydrogels

The in vitro luteolin release profiles were determined at pH 7.4 and pH 6.5 using the dialysis bag method. Composite hydrogel samples with a known luteolin content of 40 μg/mL were placed in dialysis bags (molecular weight cut-off: 8-14 kDa) and fully immersed in 20 mL of PBS at the corresponding pH. At predetermined time points, the absorbance of the release medium was measured at the maximum absorption wavelength of 355 nm using a UV-visible spectrophotometer. Luteolin concentrations were determined from a pre-established standard curve. The cumulative release rate (CRR) of luteolin was calculated as: CRR (%) = [(C_n × V_0 + Σ(C_i × V_s))/M] × 100%, where C_n is the drug concentration in the release medium at the n-th sampling, V_0 is the initial total volume of the release medium, C_i is the drug concentration at the i-th sampling, V_s is the volume taken at each sampling, and M is the total mass of luteolin loaded in the hydrogel sample.

The release profiles of bare Lut-PLGA NPs were measured under the same conditions as controls. Two-way ANOVA was used to compare the difference in release kinetics between pH 6.5 and pH 7.4.

### 2.13 Rat MI model construction and evaluation

A rat MI model was established using the classic left anterior descending (LAD) coronary artery ligation technique [26]. All animal experiments were conducted in accordance with the guidelines of the Laboratory Animal Ethics Committee of Southern Medical University and received ethical approval. Male Sprague Dawley (SD) rats aged 6 weeks and weighing 200-220 g were used. Animals were fasted for 12 h prior to surgery with free access to water. After anesthesia, a longitudinal incision was made at the 3rd-4th intercostal space to the left of the sternum, exposing the LAD coronary artery, which was ligated with a 6-0 sterile surgical suture. Animals were randomly assigned to five groups (n = 6 per group): sham-operated group, MI group, MI + GK group, MI + GK@PLGA group (GK containing blank PLGA NPs), and MI + GK@NPs group. Ten minutes after LAD ligation, a composite hydrogel cardiac patch was applied to the pale ischemic zone of the infarct area. In the sham-operated group, the chest was opened without LAD ligation.

Echocardiography was performed on postoperative days 1, 7, and 28 to evaluate cardiac function. Rats were anesthetized by isoflurane inhalation, and cardiac structure and function were non-invasively monitored using a dedicated small-animal echocardiography system. Standard parasternal long-axis and short-axis views of the left ventricle (LV) were obtained from the left sternal border. In the short-axis view, M-mode echocardiographic images were acquired at the level of the papillary muscles. All images were acquired at end-diastole and end-systole on a simultaneously recorded electrocardiogram (ECG) and stored for subsequent analysis. Cardiac function parameters were measured and calculated using the instrument's built-in software from M-mode images, with core parameters including: LV ejection fraction (LVEF, %) = [(LVEDV − LVESV)/LVEDV] × 100%; LV fractional shortening (LVFS, %) = [(LVIDd − LVIDs)/LVIDd] × 100%; LV internal diameter at end-systole (LVIDs, mm); LV internal diameter at end-diastole (LVIDd, mm); LV end-systolic volume (LVESV, μL); and LV end-diastolic volume (LVEDV, μL).

### 2.14 Histological and immunohistochemical analysis

At the experimental endpoints (days 3, 7 and 28), rats were anesthetized and sacrificed, and cardiac tissue was rapidly excised, washed in pre-chilled PBS, fixed in 4% PFA, and processed into paraffin-embedded sections. Hematoxylin and eosin (H&E) staining was used to assess pathological changes in myocardial tissue. Masson's trichrome staining was used to evaluate collagen deposition and fibrosis. Cardiomyocyte apoptosis was examined by TUNEL and cardiac troponin T (cTnT) immunofluorescence co-staining, in which cTnT labels cardiomyocytes and allows apoptosis to be quantified specifically within this population, together with cleaved caspase-3 immunofluorescence. FITC-conjugated wheat germ agglutinin (WGA) was incubated with myocardial sections to specifically label cardiomyocyte membranes for evaluation of cross-sectional area as a measure of myocardial hypertrophy. CD31/α-smooth muscle actin (α-SMA) double immunofluorescence was used to evaluate neovascularization and vessel maturation, with CD31 marking endothelial cells for capillary-density quantification and α-SMA marking vascular smooth muscle cells and activated myofibroblasts. Co-localization of CD31 and α-SMA was quantified to assess mature vessel density. Vascular endothelial growth factor (VEGF) and transforming growth factor-β (TGF-β) immunofluorescence were performed to detect the expression and distribution of these factors in myocardial tissue and to analyze their correlation with the repair process. Collagen I and Collagen III immunofluorescence co-staining was used to evaluate the deposition ratio and distribution of different collagen subtypes.

### 2.15 In vivo fluorescence tracking and transmural penetration analysis

DiR (near-infrared fluorescent probe) and luteolin were co-encapsulated into PLGA NPs via single-emulsion solvent evaporation. The NPs were physically blended into the GelMA–KerMA precursor solution and photo-crosslinked into a composite cardiac patch. In vivo fluorescence imaging was performed using an IVIS system at days 1, 3, 7, 14, and 28 post-implantation, and the cardiac-region fluorescence intensity was quantified relative to day 1. At each endpoint, the hearts were excised, embedded in OCT, cryosectioned (10 μm), counterstained with DAPI, and imaged for DiR fluorescence. The epicardium-to-endocardium DiR distribution profile was analyzed to evaluate the transmural penetration of the NPs, reflecting drug diffusion behavior.

### 2.16 Enzyme-linked immunosorbent assay (ELISA)

ELISA kits were used to analyze the expression levels of TGF-β and VEGF in myocardial tissue. Infarcted myocardial tissue from rats in each group at 28 days post-hydrogel intervention was collected and washed free of blood in pre-chilled PBS. Tissue samples were weighed and sectioned, and pre-chilled protein lysis buffer was added at a weight-to-volume ratio of 1:9. Samples were thoroughly homogenized on ice using a tissue homogenizer, and the supernatant was collected as the test sample.

### 2.17 Cardiomyocyte culture and construction of in vitro pathological models

Following previously published protocols [27], [28], H9C2 cells were cultured to 70%-80% confluency under normal conditions. They were then switched to glucose-free medium and placed in a tri-gas incubator under hypoxic conditions (37°C, 94% N_2_, 5% CO_2_, 1% O_2_) for 8 h to establish an oxygen-glucose deprivation (OGD) model. Cells were then returned to normal glucose-containing medium and normoxic conditions for indicated durations. Cell experiments were divided into five groups: control group, OGD model group, OGD + GK hydrogel extract group, OGD + GK@NPs hydrogel extract group (OGD + GK@NPs), and OGD + GK@NPs hydrogel extract + Akt inhibitor (AKTi) group (OGD + GK@NPs + AKTi). For immunofluorescence staining, cells were treated with hydrogel extracts (with or without AKTi) during the reoxygenation period, and stained for p-AKT1 and p-Bad at 4 h, or c-Casp3 at 8 h post-OGD. For transcriptomic sequencing, cells were collected at 24 h post-OGD for RNA extraction.

RCFs were serum-starved in low-serum DMEM for 12 h, and a fibrosis model was constructed following a previously published protocol [29]. Subsequently, RCFs were switched to low-serum DMEM containing 10 ng/mL recombinant human TGF-β1 and stimulated continuously at 37°C with 5% CO_2_ for 24 h. Cell experiments were divided into four groups: control group, TGF-β model group, TGF-β + GK hydrogel extract group, and TGF-β + GK@NPs hydrogel extract group.

Except for the control group, all cells received the corresponding modeling treatment. The corresponding hydrogel extracts were added concurrently with the modeling. For the OGD + GK@NPs + AKTi group, H9C2 cells were pretreated with the allosteric Akt1/2/3 inhibitor MK-2206 at 0.2 μM for 1 h before OGD and maintained throughout the OGD period. MK-2206 was dissolved in DMSO; the final DMSO concentration in all treatment groups was ≤ 0.1% (v/v) [30]. The control group received an equivalent volume of PBS.

### 2.18 In vitro antioxidant capacity assessment, cell proliferation evaluation, and mitochondrial staining

After modeling, H9C2 cell medium was discarded, and cells were labeled with 10 μM 2′,7′-dichlorofluorescein diacetate (DCFH-DA) probe. Mitochondrial superoxide was detected using the MitoSOX Red Mitochondrial Superoxide Indicator kit. Cell proliferation was assessed using the Cell-Light EdU detection kit. Mitochondrial morphology was examined in living H9C2 cells using a MitoTracker mitochondria-specific fluorescent probe according to the manufacturer's instructions. Live-cell imaging was performed using an OlyVIA confocal microscope. Mitochondrial morphology analysis was performed using ImageJ software with the MitoTracker plug-in or manual thresholding. A uniform fluorescence intensity threshold was applied to binarize the original images for individual mitochondria identification. Key quantitative parameters included mitochondrial branch length, network complexity, and degree of fragmentation.

### 2.19 Detection of cell apoptosis and mitochondrial membrane potential assessment

Apoptosis was quantified with an Annexin V-FITC/PI kit; total apoptosis was the sum of early (Annexin V-FITC^+^/PI^−^) and late (Annexin V-FITC^+^/PI^+^) rates. Mitochondrial membrane potential (MMP) was assessed with the JC-1 kit.

### 2.20 Western blot assay

H9C2 cells or RCFs were lysed in RIPA buffer with protease/phosphatase inhibitors on ice, centrifuged (12,000 × g, 30 min, 4°C), and protein concentration was measured by BCA. Equal amounts were resolved on 10% SDS-PAGE and transferred to PVDF membranes. After blocking, membranes were incubated with primary antibodies at 4°C overnight, then with horseradish-peroxidase secondary antibodies, and signals were detected by ECL. GAPDH or COX IV served as the internal reference; target expression was the target-to-reference band gray ratio.

### 2.21 Immunofluorescence staining

Treated H9C2 cells or RCFs were seeded on confocal dishes and gently washed with pre-warmed PBS. After fixation (4% PFA, 15 min) and permeabilization (0.1% Triton X-100, 5 min), cells were blocked with 5% BSA for 1 h and incubated with primary antibodies at 4°C overnight. Species-matched Alexa Fluor 488/594 secondary antibodies (1:500) and DAPI were applied before confocal imaging; fluorescence intensity was quantified in ImageJ.

### 2.22 Cell migration capacity assessment

RCFs were seeded in 6-well plates and cultured to form a dense monolayer. A straight scratch was made across the center of the cell monolayer in each well using the tip of a sterile 200 μL pipette tip. The cells were gently washed three times with PBS and divided into the following treatment groups, each with fresh low-serum DMEM basal medium: control group, TGF-β model group, TGF-β + GK extract group, and TGF-β + GK@NPs extract group. The cells were returned to a 37°C, 5% CO_2_ incubator. Images of the scratch area were captured at the same position by inverted microscopy at 0, 12, and 24 h post-scratch. The cell migration rate was calculated as: [(scratch width at 0 h − scratch width at T h)/scratch width at 0 h] × 100%.

For Transwell migration assays, we seeded 5 × 10^4^ cells in the upper chamber (200 μL serum-free medium). The lower chamber contained 600 μL of complete medium with 10% FBS, supplemented with the following test articles by group: control (medium only), GK extract group, and GK@NPs extract group. Chambers were incubated at 37°C with 5% CO_2_ for 24 h. After incubation, non-migrated cells on the upper surface were gently removed with a cotton swab. Membranes were fixed with 4% PFA for 15 min and stained with 0.1% crystal violet for 20 min. After PBS rinsing, migrated cells on the underside of the membrane were photographed in five random fields by inverted microscopy and counted.

### 2.23 In vitro angiogenesis assay

Pre-chilled 24-well plates were coated with 300 μL Matrigel per well and incubated at 37°C for 30 min to allow complete polymerization. Simultaneously, HUVECs were digested, counted, and resuspended in serum-free DMEM containing GK or GK@NPs extracts. Cells (2 × 10^4^ per well) were seeded onto the polymerized Matrigel and returned to the incubator for 6 h. Tube morphology was observed by inverted microscopy. The formed tubular network, including total tube length, number of meshes, and number of branch points, was quantified using ImageJ software with the Angiogenesis Analyzer plug-in.

### 2.24 mRNA sequencing analysis

H9C2 cells from the OGD model group and the OGD + GK@NPs treatment group were lysed with TRIzol reagent, and total RNA was extracted according to the manufacturer's instructions. mRNA was purified from total RNA using a column-based enrichment method and used as the starting material for reverse transcription and high-throughput RNA sequencing.

### 2.25 Statistical analysis

All quantitative data are presented as mean ± standard deviation. Statistical analyses were performed using GraphPad Prism 10.6.1 software. Comparisons between two groups were performed using an unpaired, two-tailed Student's t-test. For comparisons among multiple groups, one-way analysis of variance (ANOVA) followed by Tukey's multiple comparisons post-hoc test was used to determine specific pairwise differences. Two-way ANOVA was adopted for data with two independent variables, including time and pH (for in vitro release) or time and myocardial region (for in vivo frozen sections). For longitudinal data repeatedly measured in the same animals, two-way repeated-measures ANOVA was applied. All post-hoc pairwise comparisons were conducted using Tukey's multiple comparisons test. Images were processed using ImageJ software. A *P* value < 0.05 was considered statistically significant.

## 3 Results

### 3.1 Preparation and characterization of luteolin-loaded PLGA nanoparticles

Luteolin-loaded PLGA nanoparticles were prepared by the classical O/W emulsion-solvent evaporation (Fig. 1a). Zeta potential analysis showed that blank PLGA nanoparticles had a potential of −8.26 mV, which decreased significantly to −28.0 mV after luteolin loading, suggesting increased surface electronegativity (Fig. 1b). Particle size and zeta potential measurements revealed that blank PLGA nanoparticles had a diameter of approximately 140 nm; upon loading luteolin, the diameter of Lut-PLGA NPs increased to approximately 200 nm, with a uniform size distribution consistent with the expected range for nanoscale drug delivery systems (Fig. 1c). The UV-Vis spectrum of luteolin and the corresponding calibration curve are presented in Supplementary Fig. 1. The EE and DL of luteolin were determined to be (84.1 ± 2.6)% and (5.35 ± 0.28)%, respectively (Supplementary Table 1).

**Fig. 1:**
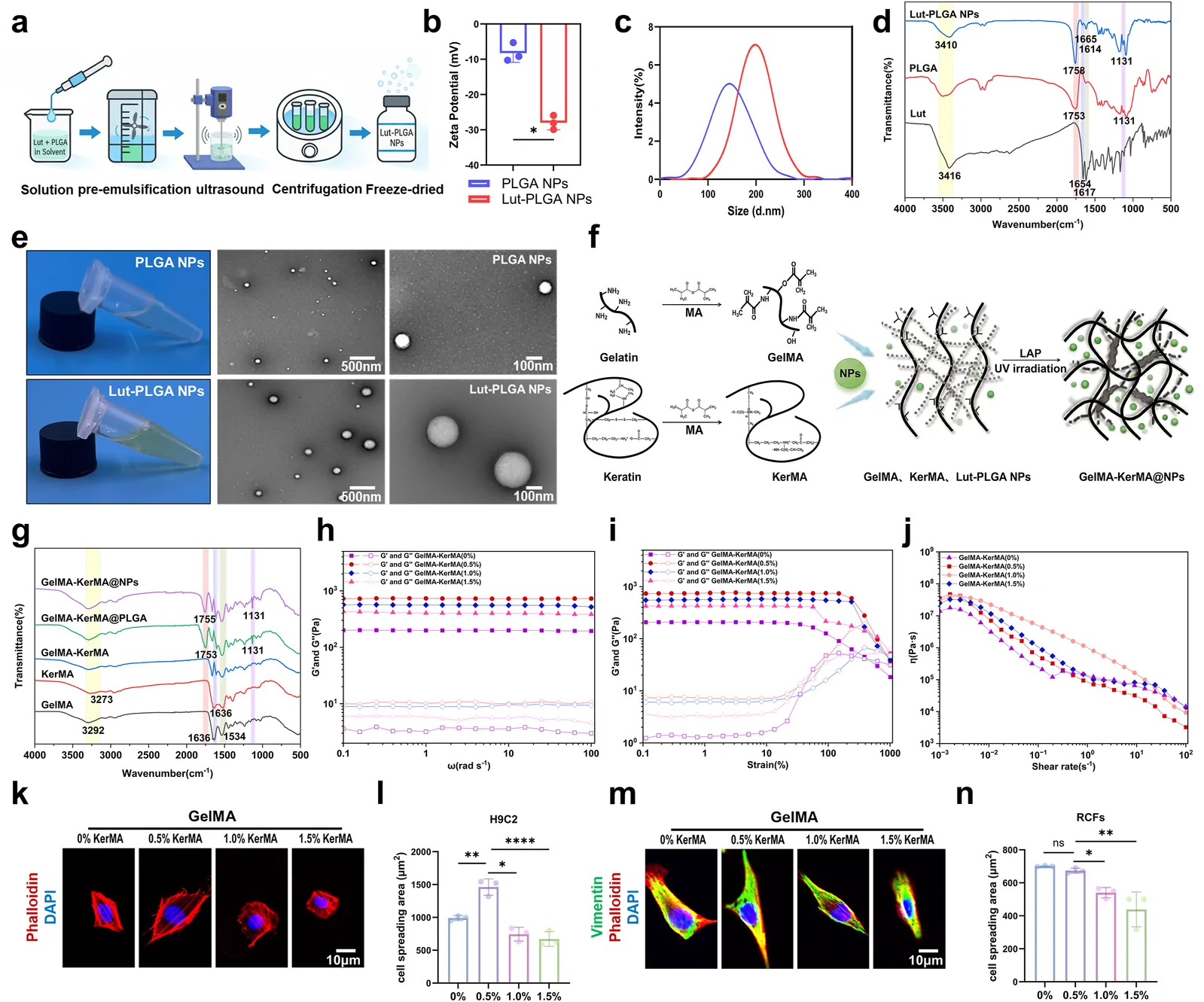
Preparation and basic characterization of Lut-PLGA nanoparticles and GK composite hydrogel. (a) Schematic illustration of Lut-PLGA NP preparation by the O/W emulsion method. (b) Zeta potential measurements of blank PLGA NPs and Lut-PLGA NPs (n = 3, data presented as mean ± SD, *P < 0.05). (c) Particle size distribution curves measured by DLS. (d) FTIR spectra of pure luteolin, PLGA, and Lut-PLGA NPs. (e) Digital photograph and TEM images of NPs (scale bars: 500 nm and 100 nm). (f) Schematic illustration of photo-crosslinked synthesis of GK@NPs. (g) FTIR spectra comparison of various hydrogel materials. (h) Frequency sweep results of hydrogels with different KerMA ratios: fixed strain 1%, frequency range 0.1–10 Hz. (i) Amplitude sweep results: fixed frequency 1 Hz, strain range 0.1%–100%. (j) Viscosity sweep results: shear rate 0.1–100 s^−1^. (k,l) Fluorescence staining of H9C2 cardiomyocytes cultured on hydrogels for 24 h (k, F-actin cytoskeleton in red and DAPI nuclear staining in blue) and quantitative analysis of H9C2 cell spreading area (l). (m,n) Fluorescence staining of RCFs cultured on hydrogels for 24 h (m, Vimentin in green, F-actin cytoskeleton in red, and DAPI nuclear staining in blue) and quantitative analysis of RCF cell spreading area (n). (Data presented as mean ± SD, n = 3, ns: no significant differences, *P < 0.05, **P < 0.01, ***P < 0.001, ****P < 0.0001). (For interpretation of the references to colour in this figure legend, the reader is referred to the Web version of this article.)

To further verify successful luteolin encapsulation and the interactions within the nanoparticles, FTIR spectroscopy was used to analyze the chemical structures of the materials (Fig. 1d). Pure luteolin displayed characteristic phenolic hydroxyl and benzene ring skeletal vibration peaks at 3416 cm^−1^, 1654 cm^−1^, and 1617 cm^−1^. Blank PLGA exhibited characteristic ester carbonyl and C-O-C stretching vibration peaks at 1753 cm^−1^ and 1131 cm^−1^. The spectrum of Lut-PLGA NPs showed characteristic signals from both PLGA and luteolin, confirming successful complexation of the drug and carrier. Notably, the characteristic phenolic hydroxyl peak of luteolin shifted from 3416 cm^−1^ to 3410 cm^−1^, suggesting hydrogen bond formation between luteolin and the PLGA polymer chains that may contribute to sustained drug release. TEM confirmed spherical morphology with uniform size and clear boundaries (Fig. 1e).

### 3.2 Preparation and characterization of GK composite hydrogels

GelMA and KerMA photo-sensitive monomers were separately prepared by methacrylic anhydride modification. Using LAP as a photoinitiator, UV irradiation triggered free-radical polymerization of the methacryloyl double bonds to form the GK composite hydrogel (Fig. 1f). To identify a suitable matrix formulation for myocardial repair, the GelMA concentration was fixed at 5% (w/v), and KerMA was incorporated at gradient ratios. This yielded three composite systems (GK-0.5, GK-1.0, and GK-1.5) for systematic investigation of how KerMA content modulates the microstructure, mechanical stability, cellular compatibility, and biocompatibility of the hydrogel.

FTIR results showed (Fig. 1g) that pure GelMA, KerMA, and the composite hydrogel all displayed protein-characteristic amide I bands at 1636 cm^−1^ (C=O stretching vibration of peptide bonds). Compared with single components, the amide I band of the GK composite hydrogel was markedly broadened, with asymmetric shoulders appearing in the 1620-1640 cm^−1^ region. This phenomenon is attributed to the formation of extensive intermolecular hydrogen bonds between GelMA and KerMA molecular chains.

Rheological performance is a core indicator for evaluating the mechanical stability and dynamic cardiac adaptability of cardiac patches. Frequency sweep results showed that the storage modulus (G′) remained significantly higher than the loss modulus (G″) across the entire test range for all hydrogel groups, demonstrating stable elastic solid characteristics and a mechanical basis for sustained support of the infarcted ventricular wall against ventricular deformation (Fig. 1h). Strain sweep results indicated that the GK-0.5 group exhibited a wide linear viscoelastic region (LVR), conferring greater structural deformation tolerance and mechanical stability (Fig. 1i). All hydrogel groups also exhibited typical shear-thinning behavior (Fig. 1j). Viscosity reversibly decreases under shear force. This reversible shear-thinning behavior supports injectability and intraoperative shaping for minimally invasive implantation.

Cell adhesion and spreading results showed that, compared with GelMA alone, the addition of appropriate amounts of KerMA significantly improved scaffold biocompatibility and the adhesion and growth behavior of cardiomyocytes and fibroblasts (Fig. 1k–n). The GK-0.5 group demonstrated a favorable spreading morphology and an increased spreading area for both H9C2 cardiomyocytes and RCFs, reaching (1462 ± 124) μm^2^ and (673.6 ± 15.2) μm^2^, respectively. These results indicate that low-dose KerMA incorporation can effectively simulate the native myocardial extracellular matrix (ECM) microenvironment, facilitating cell anchoring, extension, and growth. In contrast, further increasing KerMA content to 1.0% (GK-1.0) and 1.5% (GK-1.5) significantly reduced the spreading area of both H9C2 cardiomyocytes and RCFs. Cells exhibited a more rounded morphology with fewer lamellipodia, indicating that excessive KerMA incorporation impairs, rather than promotes, cell–material interactions. This non-monotonic trend suggests an optimal KerMA dosage for supporting cell spreading. Considering the combined rheological performance (widest LVR), spreading area, and absence of cytotoxicity (all *P* > 0.05, Fig. 2h and i), GK-0.5 was selected as the preferred matrix formulation for subsequent loading of Lut-PLGA NPs to construct the pH-responsive cardiac patch.

**Fig. 2:**
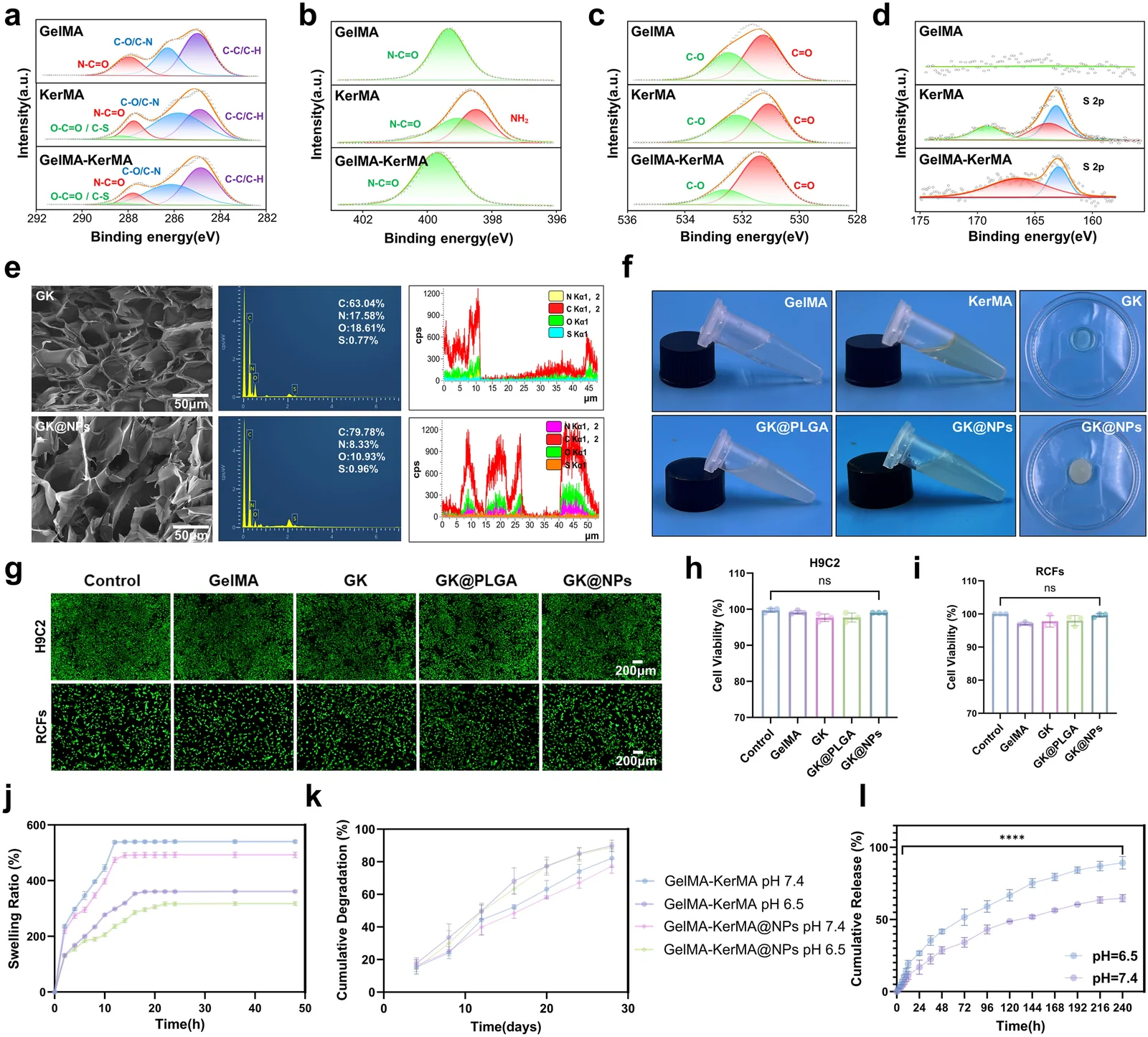
Chemical composition, microstructure, biocompatibility and pH-responsive properties of composite hydrogels. (a-d) High-resolution XPS spectra of the prepared hydrogels (C 1s, N 1s, O 1s, S 2p). (e) SEM images and EDS elemental analysis of GK and GK@NPs. (f) Macroscopic appearance of different hydrogel samples. (g) Live/dead fluorescence staining of H9C2 cardiomyocytes and RCFs cultured in hydrogel extracts; live cells stained green by Calcein-AM and dead cells stained red by PI. (h, i) Cell viability assessed by CCK-8 assay. (j, k) Swelling curves (j) and cumulative degradation curves (k) of the GK matrix and GK@NPs at pH 7.4 and pH 6.5. (l) Cumulative luteolin release profiles of GK@NPs at pH 7.4 and pH 6.5. ****P < 0.0001 by two-way ANOVA for the overall effect of pH. (Data presented as mean ± SD, n = 3, ns: no significant differences, *P < 0.05, **P < 0.01, ***P < 0.001, ****P < 0.0001). (For interpretation of the references to colour in this figure legend, the reader is referred to the Web version of this article.)

FTIR and XPS were employed to analyze interactions between the two components and the material's chemical properties. XPS survey scans (Fig. 2a–d, Supplementary Fig. 2) detected a characteristic S 2p signal attributable to KerMA, confirming uniform complexation of both polymeric components. High-resolution N 1s spectra revealed that pure KerMA exhibited a characteristic sub-peak at 398.5 eV corresponding to free primary amine groups (-NH_2_). Upon composite hydrogel formation, this sub-peak completely disappeared, and the main nitrogen peak shifted toward lower binding energy. This change is attributed to the formation of strong intermolecular hydrogen bonds between the free amino groups of KerMA and the carbonyl/hydroxyl groups on the GelMA chains. Together with the physical shielding effect of the photo-crosslinked network, this further reduces the surface accessibility of the amino groups, collectively leading to the redistribution of the N 1s signals.

SEM and EDS results demonstrated that the GK@NPs composite hydrogel formed an interconnected 3D porous network with a uniform pore size distribution in the 50-100 μm range. This biomimetic porous structure facilitates nutrient diffusion, provides ample space for cell adhesion and migration, and supports inward growth of nascent blood vessels. EDS clearly detected uniform distribution of the KerMA-characteristic sulfur element throughout the hydrogel network (Fig. 2e), confirming homogeneous dispersion of the keratin component without local aggregation, thus ensuring uniformity of overall material performance. All GK hydrogels formed transparent, uniform patches (Fig. 2f).

Good cytocompatibility is a prerequisite for biomaterials intended for in vivo myocardial repair. Live/dead staining results showed negligible proportions of dead cells across all hydrogel formulation groups, with all groups maintaining high cell viability (Fig. 2g). Quantitative CCK-8 results further confirmed that there were no statistically significant differences in H9C2 and RCF cell viability among hydrogels of different KerMA ratios (*P* > 0.05), with no concentration-dependent cytotoxicity observed (Fig. 2h and i). These results indicate that the GK composite system has good and consistent biocompatibility, adequately meeting the requirements for subsequent experiments.

### 3.3 pH-responsive properties and drug release behavior of GK@NPs hydrogels

Rheological testing confirmed that the composite hydrogels exhibited stable gel characteristics under both pH conditions (Supplementary Fig. 3a and 3b). Under acidic conditions, the storage modulus showed a modest, reversible decrease, indicating pH-dependent mechanical compliance rather than network breakdown. The hydrogel features a dual network of covalent crosslinks and hydrogen bonds. The GelMA and KerMA molecular chains contain ionizable carboxyl and amino groups whose protonation state changes with pH, thereby modulating interchain electrostatic repulsion and reversible swelling/compliance. At pH 7.4, deprotonation strengthens electrostatic repulsion, causing network expansion and higher swelling ratios; at pH 6.5, protonation reduces this repulsion and enhances intermolecular hydrogen bonding, producing a more compact, lower-swelling state (Fig. 2j) and a modest, reversible decrease in storage modulus (Supplementary Fig. 3a and 3b). This charge-driven conformational change alters mesh size and diffusion resistance but does not break the permanent covalent network and therefore does not itself cause degradation. As shown in Fig. 2k, both the GK matrix and GK@NPs underwent gradual cumulative mass loss over 28 days, with a somewhat greater extent at pH 6.5 than at pH 7.4. This long-term bulk erosion reflects slow hydrolysis of the protein network and gradual particle release through slow surface erosion over weeks, rather than pH-triggered rapid dissociation of the covalent backbone. Particle erosion generates local voids that facilitate drug diffusion, while the covalent GelMA–KerMA network remains macroscopically intact over the therapeutic window. The resulting modest softening improves compliance with the ischemic myocardium without compromising structural support.

In vitro drug release results (Fig. 2l, Supplementary Fig. 4) showed that at pH 6.5, the cumulative release rates were significantly higher than those at pH 7.4. Supplementary Fig. 4 shows the release profiles of bare Lut-PLGA nanoparticles under the same conditions. Bare Lut-PLGA NPs exhibited mean cumulative release rates of (92.4 ± 0.3)% at 144 h at pH 6.5 and (79.6 ± 2.7)% at pH 7.4. When nanoparticles were incorporated into the GK matrix, drug release was overall retarded. GK@NPs displayed mean cumulative release values of (89.2 ± 4.4)% at 240 h at pH 6.5 and (64.7 ± 2.6)% at pH 7.4. Two-way ANOVA confirmed a highly significant overall effect of pH on the cumulative release profiles of both bare nanoparticles and composite hydrogels (both *P* < 0.0001), indicating pH-responsive release. Notably, under acidic conditions the hydrolysis rate of PLGA ester bonds increases, accelerating release of encapsulated luteolin; this acid-catalyzed PLGA hydrolysis is the primary driver of accelerated release, whereas the covalent network mainly retards and sustains release rather than triggering it, yielding faster release in the acidic infarct lesion and slower, sustained release in normal tissue. It should be noted that the dialysis bag method introduces an additional diffusion barrier and serves as a standard in vitro model for relative comparison of release kinetics under different pH conditions [31]. The initial burst release within 48 h overlaps the acute acidic phase after MI [32], while the sustained rise over 240 h corresponds to the subacute-to-chronic remodeling phase, during which inflammation and ECM turnover persist. This staged profile aligns with the prolonged post-MI acidic window rather than a transient pH pulse: acidosis develops over hours and persists over days to weeks, and cumulative acid-catalyzed PLGA hydrolysis proceeds throughout this period, while the network retards release. However, direct in vivo release kinetics were not measured in this study.

The hemolysis assay is a critical index of in vivo safety for implantable cardiac patches (Supplementary Fig. 5). In this study, the hemolysis ratios of all tested materials were below the 5% biosafety threshold, with no statistically significant differences. No erythrocyte lysis was observed, indicating acceptable blood compatibility of the composite hydrogel and meeting the in vivo biosafety requirements for orthotopic patch implantation in the rat MI model.

### 3.4 In vivo cardiac function assessment

A schematic of the in vivo experimental design is shown in Fig. 3a. Briefly, the rat MI model was established by LAD coronary artery ligation. Blank GK hydrogel or drug-loaded composite hydrogel was applied to the infarct area. The sham-operated group served as control. Cardiac function was assessed by echocardiography on postoperative days 1, 7 and 28 (Fig. 3b). Echocardiographic assessment on day 1 post-surgery showed no significant differences in LVEF and LVFS among the MI, MI + GK, MI + GK@PLGA, and MI + GK@NPs groups, confirming uniform infarct severity and comparable baseline cardiac function across all experimental groups (Supplementary Fig. 6). Quantitative analysis of echocardiographic parameters demonstrated that cardiac function was significantly impaired in the MI model group, manifested as markedly reduced LVEF and LVFS at days 7 and 28 (Fig. 3c and d). Simultaneously, LVEDV, LVESV, LVIDs, and LVIDd were all significantly increased in the MI group, indicating ventricular dilation and remodeling (Fig. 3e–h). Hydrogel treatments improved cardiac function to varying degrees. Notably, compared with the MI + GK group and the MI + GK@PLGA group, the MI + GK@NPs group exhibited significantly higher LVEF and LVFS and lower ventricular volumes and internal diameters.

**Fig. 3:**
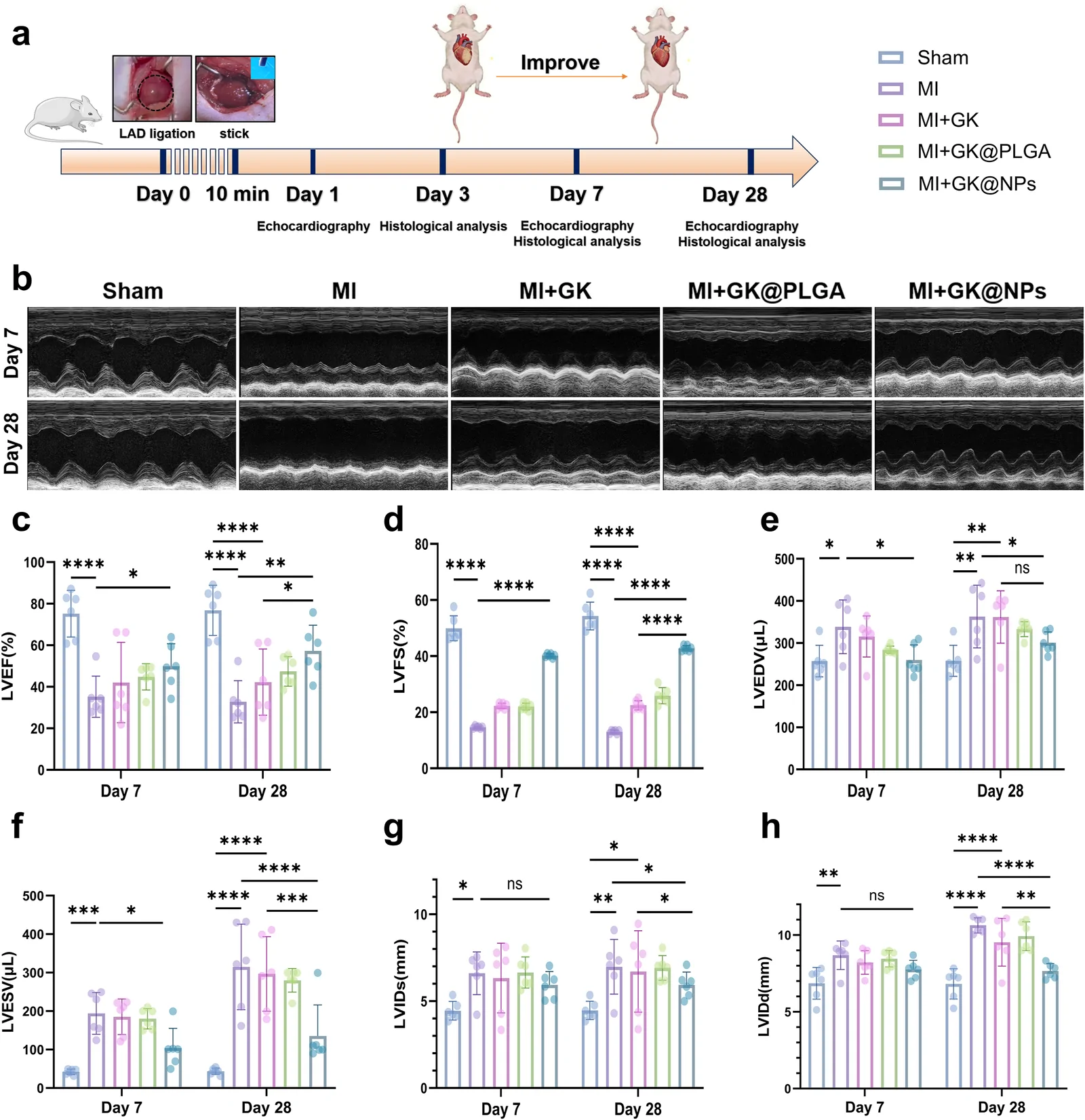
In vivo experimental design and cardiac function assessment. (a) Schematic diagram of rat MI model establishment and treatment and sampling timeline. (b) Representative echocardiographic images of rat hearts from different groups at postoperative day 7 and day 28. (c–h) Quantitative analysis of echocardiographic parameters: (c) LVEF, (d) LVFS, (e) LVEDV, (f) LVESV, (g) LVIDs, (h) LVIDd. (Data presented as mean ± SD, n = 6, ns: no significant differences, *P < 0.05, **P < 0.01, ***P < 0.001, ****P < 0.0001).

### 3.5 Anti-apoptotic, anti-fibrotic, and pro-angiogenic effects of composite hydrogel in vivo

For the assessment of myocardial injury and fibrosis, histological analysis of cardiac sections was performed at day 28. H&E staining revealed extensive cardiomyocyte necrosis, inflammatory cell infiltration, and structural disarray in the MI group. The MI + GK group and the MI + GK@PLGA group showed partial improvement, while the MI + GK@NPs group demonstrated relatively better myocardial structural recovery with reduced necrotic areas (Fig. 4a). Quantitative analysis of ventricular wall thickness further confirmed the structural recovery of the infarcted myocardium (Fig. 4b). Masson's trichrome staining showed that the collagen volume fraction (CVF) in the infarct area was (37.2 ± 8.4)% in the MI group, decreasing to (11.9 ± 2.8)% in the MI + GK@NPs group; quantitative analysis confirmed effective inhibition of myocardial fibrosis (Fig. 4a and c). WGA staining was performed to assess cardiomyocyte cross-sectional area. The MI group displayed cardiomyocyte hypertrophy, which was significantly reduced in the MI + GK@NPs group (Fig. 4d and g).

**Fig. 4:**
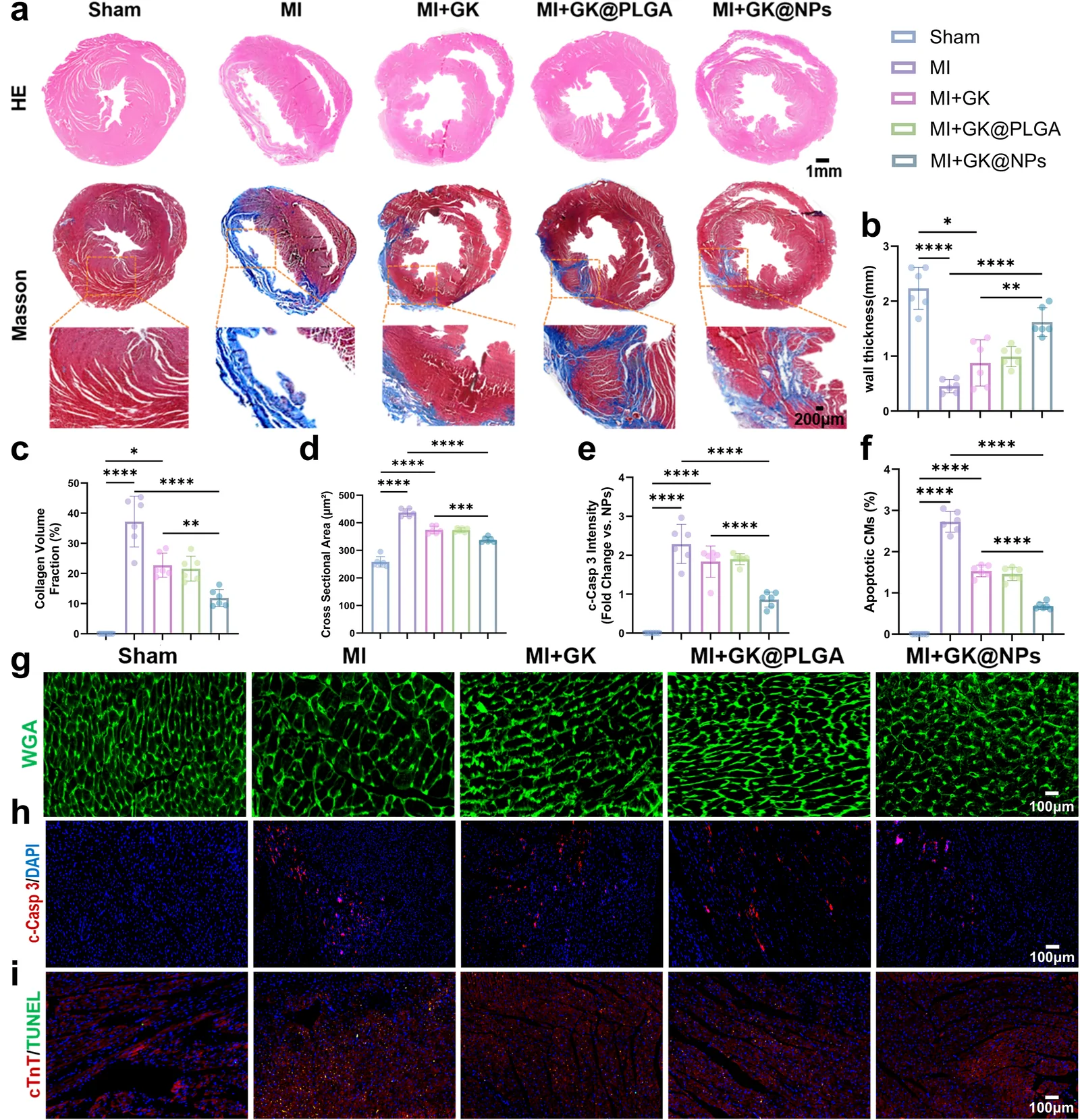
Histopathological analysis and apoptosis detection in myocardial tissue. (a) H&E and Masson's trichrome staining of cardiac cross-sections at postoperative day 28. (b) Quantitative analysis of ventricular wall thickness. (c) Quantitative analysis of CVF. (d) Quantitative analysis of cardiomyocyte cross-sectional area. (e) Quantitative analysis of c-Casp3 fluorescence intensity at day 3. (f) Quantitative analysis of apoptotic cardiomyocytes at day 3. (g) WGA fluorescence staining of cardiomyocytes (day 28). (h) Immunofluorescence staining of c-Casp3 at day 3. (i) TUNEL and cTnT co-immunofluorescence staining for apoptotic cardiomyocytes at day 3. (Data presented as mean ± SD, n = 6, *P < 0.05, **P < 0.01, ***P < 0.001, ****P < 0.0001).

At day 3 post-MI, within the acute phase of infarction, cleaved caspase-3 immunofluorescence staining showed strong positive signals in the MI group; hydrogel treatment, particularly in the drug-loaded group, significantly reduced cleaved caspase-3 expression (Fig. 4e and h). The MI group showed abundant TUNEL-positive apoptotic cardiomyocytes, with an apoptotic cell ratio of (2.72 ± 0.25)%. Compared with the MI group, the apoptotic cell ratio decreased to (1.53 ± 0.14)% in the MI + GK group and to (1.46 ± 0.16)% in the MI + GK@PLGA group, and was further reduced to (0.68 ± 0.09)% in the MI + GK@NPs group (Fig. 4f and i, Supplementary Fig. 8a). Only TUNEL^+^/cTnT^+^ double-positive cells were counted; TUNEL^+^/cTnT^−^ non-cardiomyocytes were excluded. These results collectively confirmed the anti-apoptotic effect of the composite hydrogel.

Immunofluorescence co-staining of α-SMA (marker of vascular smooth muscle cells and activated myofibroblasts) and CD31 (endothelial cell marker) was performed to evaluate neovascularization and vessel maturation in the infarct area (Fig. 5a, Supplementary Fig. 8b). The MI group exhibited the lowest α-SMA/CD31 positive density, indicating impaired angiogenesis. Hydrogel treatments promoted new vessel formation; only α-SMA signals ensheathing CD31^+^ endothelial tubes (perivascular vascular smooth-muscle/pericyte coating) were counted as mature vessels, whereas interstitial non-perivascular α-SMA was not used as a fibrosis metric (Fig. 5d). The MI + GK@NPs group showed the highest density of such CD31^+^/α-SMA^+^ mature vessels.

**Fig. 5:**
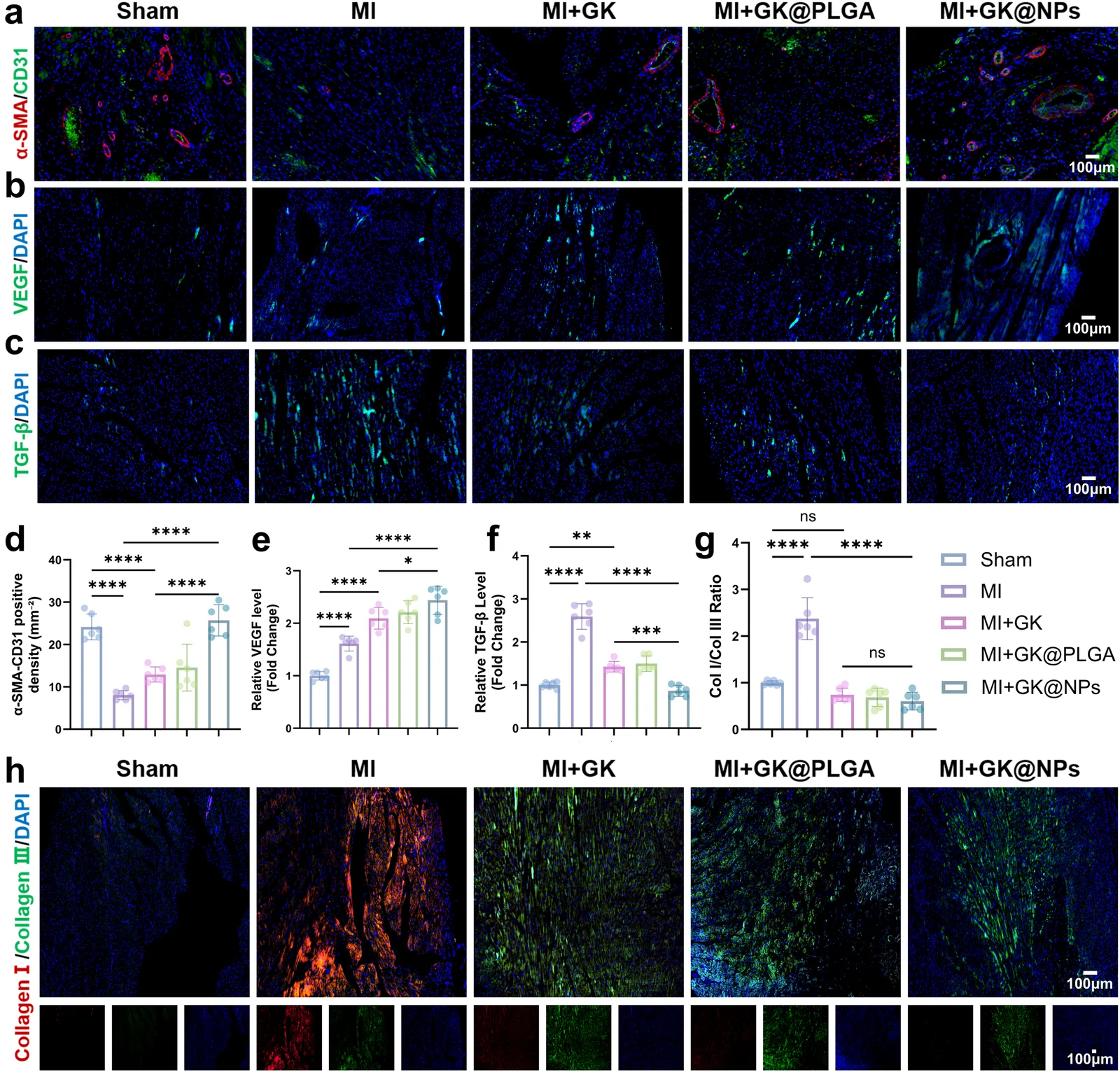
Assessment of angiogenesis- and fibrosis-related markers in myocardial tissue. (a) Immunofluorescence co-staining of α-SMA and CD31. (b) Immunofluorescence staining of VEGF. (c) Immunofluorescence staining of TGF-β. (d) Quantitative analysis of α-SMA/CD31 double-positive mature vessel density. (e) Quantitative analysis of relative VEGF expression. (f) Quantitative analysis of relative TGF-β expression. (g) Quantitative analysis of Collagen I/Collagen III ratio. (h) Immunofluorescence co-staining of Collagen I and Collagen III. (Data presented as mean ± SD, n = 6, ns: no significant differences, *P < 0.05, **P < 0.01, ***P < 0.001, ****P < 0.0001).

To evaluate the molecular regulation of angiogenesis and fibrosis, VEGF and TGF-β expression levels were examined. Immunofluorescence staining and ELISA results demonstrated that VEGF expression was significantly upregulated in hydrogel treatment groups, with the MI + GK@NPs group showing the highest levels (Fig. 5b and e, Supplementary Fig. 9c). Conversely, the pro-fibrotic factor TGF-β was markedly increased in the MI group but reduced in the hydrogel-treated groups, particularly in the MI + GK@NPs group (Fig. 5c and f, Supplementary Fig. 9b). Immunofluorescence co-staining for Collagen I and Collagen III further demonstrated excessive collagen deposition in the MI group, which was significantly reduced in the MI + GK@NPs group, confirming the anti-fibrotic effect of the composite hydrogel (Fig. 5g and h). In vivo tracking of DiR, co-encapsulated with luteolin in the same PLGA NPs as a carrier surrogate, showed sustained retention and an epicardium-to-endocardium fluorescence profile across the ischemic wall (Supplementary Fig. 7); this maps carrier penetration rather than directly measuring local luteolin concentration.

Finally, to assess the systemic biocompatibility of the composite hydrogel, major organs, including the lung, liver, spleen, and kidney, were collected for histological analysis at day 28 following cardiac perfusion. H&E staining revealed no obvious pathological changes, inflammatory infiltration, or organ damage in any hydrogel treatment group, indicating favorable in vivo biocompatibility of the prepared composite hydrogel (Supplementary Fig. 9a).

### 3.6 Protective effects of composite hydrogels on H9C2 cardiomyocytes against OGD injury

Live/dead fluorescence staining was used to observe cell survival. The control group maintained a high proportion of viable cells, whereas the OGD model group exhibited marked cell death. Importantly, clearly increased cell survival was observed in the GK@NPs group, which was corroborated by quantitative CCK-8 assay (Fig. 6a and e).

**Fig. 6:**
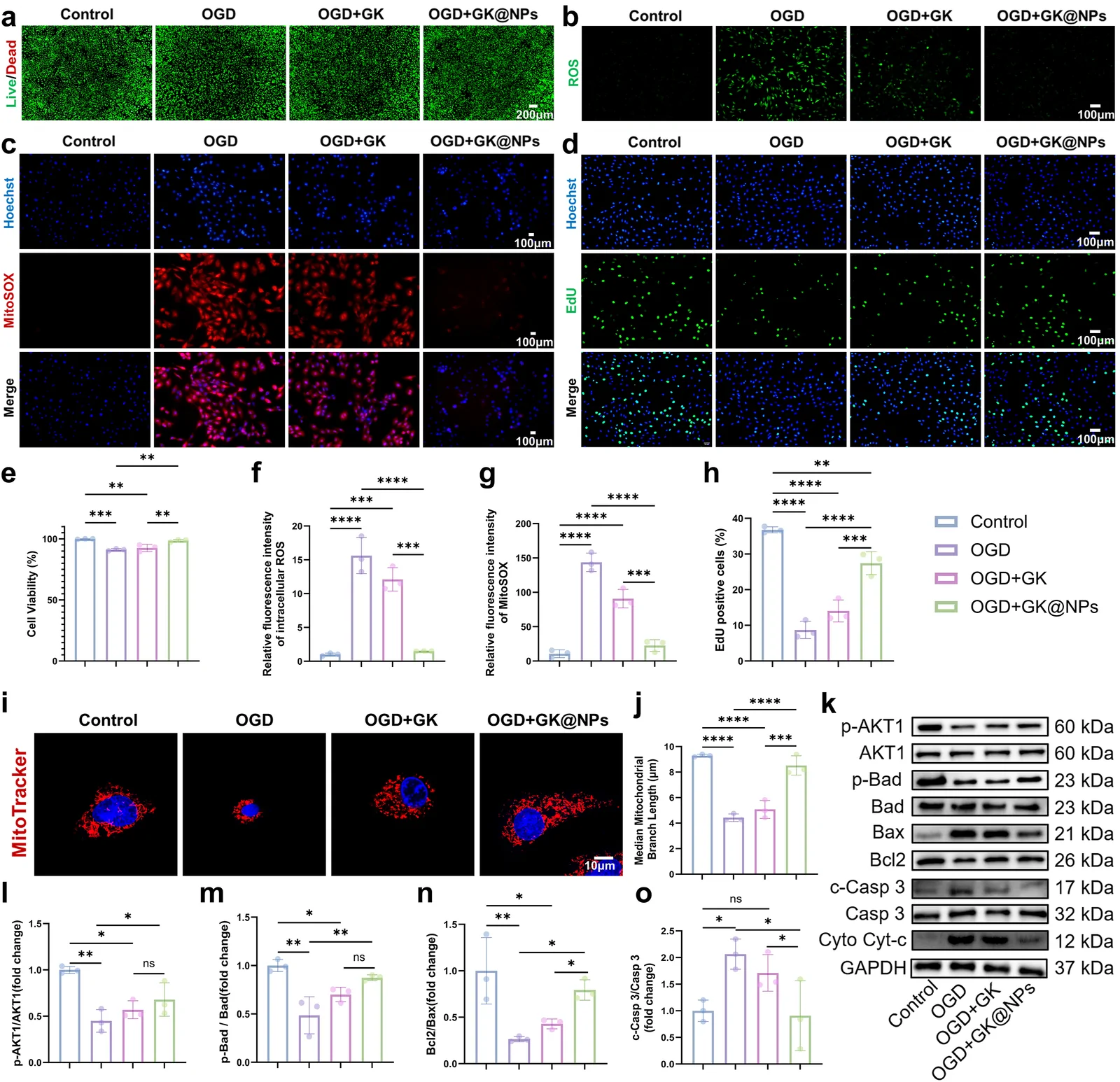
Protective effects of composite hydrogels on OGD-injured H9C2 cardiomyocytes. (a) Live/dead fluorescence staining of H9C2 cells (Calcein-AM labels live cells green; PI labels dead cells red). (b) ROS fluorescence staining. (c) MitoSOX and Hoechst staining. (d) EdU and Hoechst staining. (e) Quantitative analysis of cell viability. (f) Quantitative analysis of relative intracellular ROS fluorescence intensity. (g) Quantitative analysis of relative MitoSOX fluorescence intensity. (h) Quantitative analysis of EdU-positive cell ratio. (i) MitoTracker staining for mitochondrial morphology. (j) Quantitative analysis of median mitochondrial branch length. (k) Western blot analysis of apoptosis- and Akt/Bad pathway-related proteins. (l–o) Quantitative analysis of relative protein expression: (l) p-AKT1/AKT1, (m) p-Bad/Bad, (n) Bcl-2/Bax, (o) c-Casp3/Casp3. (Data presented as mean ± SD, n = 3, ns: no significant differences, *P < 0.05, **P < 0.01, ***P < 0.001, ****P < 0.0001). (For interpretation of the references to colour in this figure legend, the reader is referred to the Web version of this article.)

Intracellular ROS fluorescence intensity was markedly elevated in OGD-stimulated H9C2 cells. GK@NPs treatment not only partially scavenged excess ROS but also exerted stronger antioxidant activity, thereby effectively alleviating oxidative stress (Fig. 6b and f). Mitochondrial oxidative stress and morphological integrity were further evaluated. MitoSOX staining showed that OGD exposure significantly increased mitochondrial superoxide levels. Hydrogel formulations effectively reduced mitochondrial superoxide accumulation, with the OGD + GK@NPs group exhibiting the lowest relative MitoSOX fluorescence intensity (Fig. 6c and g). Cell proliferative activity was assessed by EdU incorporation. The hypoxic microenvironment significantly suppressed cardiomyocyte proliferative capacity. Hydrogel treatment effectively restored inhibited cell growth, with the highest percentage of EdU-positive proliferating cells detected in the GK@NPs group (Fig. 6d and h). The OGD group exhibited severe mitochondrial fragmentation, which was effectively rescued by hydrogel intervention. Cells treated with drug-loaded hydrogel retained a more complete and ordered mitochondrial network (Fig. 6i and j).

To elucidate the underlying molecular mechanism, Western blot analysis was performed to examine the intrinsic mitochondrial apoptosis pathway. Compared with the normal control group, OGD injury upregulated the expression of pro-apoptotic proteins, including Bax, cleaved caspase-3, and cytosolic cytochrome *c*, while concurrently downregulating the anti-apoptotic protein Bcl-2. In the GK@NPs group, the Bax/Bcl-2 ratio and cytochrome *c* release from mitochondria were both significantly reduced, thereby blocking progression of the intrinsic apoptotic cascade (Fig. 6k and 6l-o). Mitochondrial cytochrome *c* level was further normalized to the mitochondrial marker COX IV (Supplementary Fig. 11a–g), which consistently confirmed reduced cytochrome *c* release from mitochondria upon hydrogel treatment.

### 3.7 Transcriptomic analysis of GK@NPs-Treated H9C2 cardiomyocytes

Differentially expressed genes (DEGs) were identified by comparing the transcriptomic profiles of the OGD group and the OGD + GK@NPs group. The volcano plot (Fig. 7a) visualized the distribution of DEGs, revealing that 516 genes were significantly upregulated or downregulated following drug-loaded hydrogel treatment. The hierarchical clustering heatmap (Supplementary Fig. 12a) further confirmed distinctly different gene expression patterns between the two groups, indicating that the composite hydrogel induced profound transcriptional responses.

**Fig. 7:**
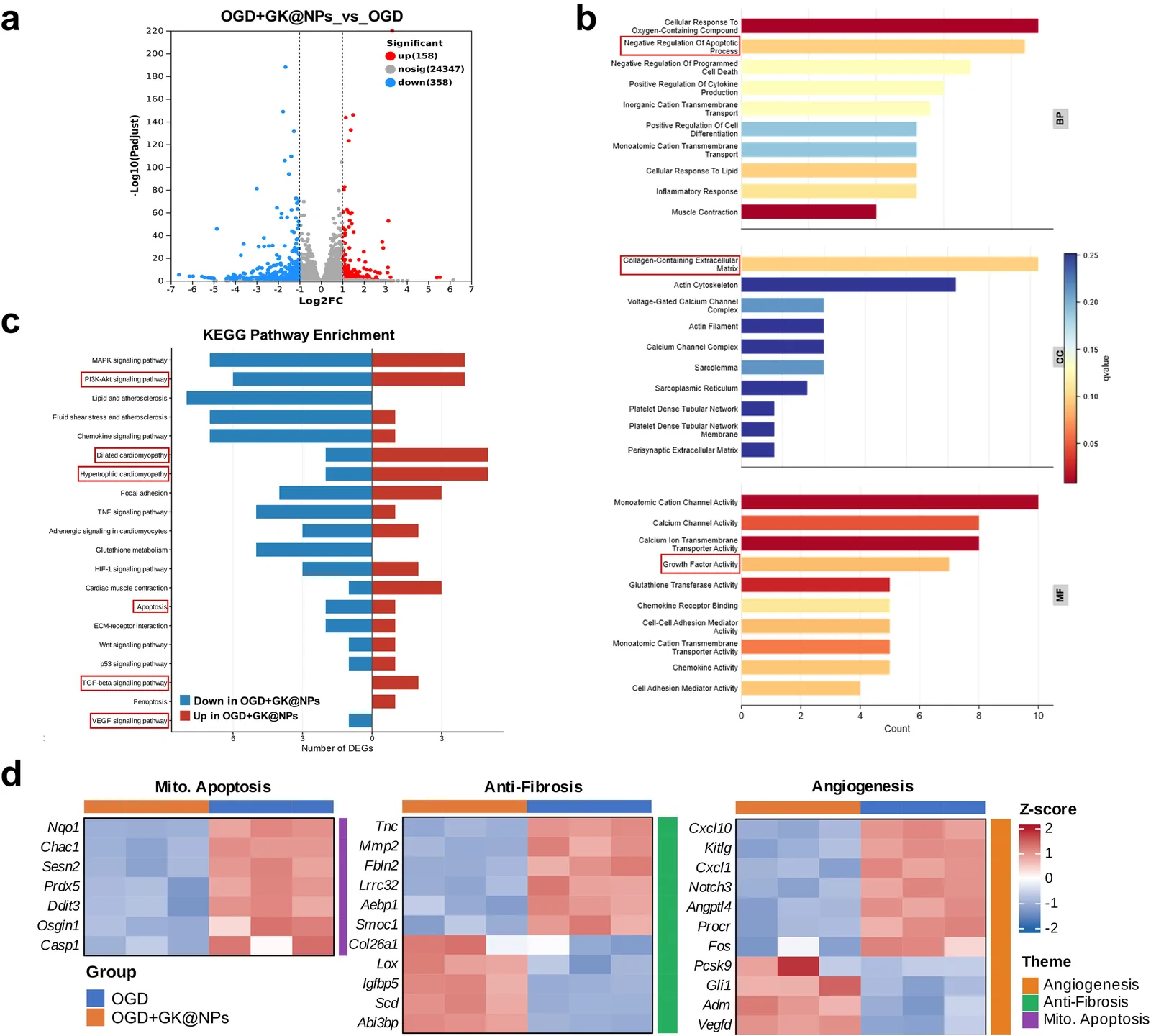
Transcriptomic sequencing analysis of H9C2 cardiomyocytes. (a) Volcano plot of DEGs between the OGD group and the OGD + GK@NPs group; red and blue dots represent significantly upregulated and downregulated genes, respectively (|log_2_ fold change| > 1, adjusted *P* < 0.05). (b) GO functional enrichment analysis. (c) Bar chart of KEGG pathway enrichment statistical distribution. (d) Heatmap of representative DEGs categorized into three functional groups: mitochondrial apoptosis (*Casp1*, *Chac1*, *Sesn2*, etc.), anti-fibrosis (*Tnc*, *Mmp2*, etc.), and angiogenesis (*Cxcl10*, *Kitlg*, etc.). (For interpretation of the references to colour in this figure legend, the reader is referred to the Web version of this article.)

Gene Ontology (GO) functional enrichment analysis was performed to annotate biological processes associated with DEGs. As shown in Fig. 7b and Supplementary Fig. 12c, the enriched biological processes were primarily related to cellular stress responses, negative regulation of apoptosis, and ECM organization. Specifically, key terms such as “negative regulation of apoptotic process” and “cellular response to oxygen-containing compounds” were significantly enriched, consistent with the anti-apoptotic phenotype observed in our cellular experiments.

Kyoto Encyclopedia of Genes and Genomes (KEGG) pathway enrichment analysis (Fig. 7c and Supplementary Fig. 12b) further highlighted several key signaling pathways, including the MAPK signaling pathway and the Rap1 signaling pathway, both known to be closely associated with cell survival, apoptosis, and stress responses. Notably, the Akt signaling pathway was not among the top enriched pathways. This is attributable to the fact that Akt activation is dominated by rapid post-translational phosphorylation (peaking at 4 h) rather than transcriptional regulation. Our RNA-seq was performed at 24 h post-OGD, which mainly captures late-stage transcriptional changes driven by sustained MAPK/Rap1 signaling rather than early phosphorylation events.

To further link the transcriptomic data with the observed biological effects, DEGs were categorized into three functional groups: mitochondrial apoptosis, anti-fibrosis, and angiogenesis. As shown in Fig. 7d, several core genes related to the mitochondrial apoptosis pathway, including *Casp1*, *Chac1*, and *Sesn2*, were significantly regulated by hydrogel treatment. Key genes involved in anti-fibrosis (*Tnc*, *Mmp2*) and angiogenesis (*Cxcl10*, *Kitlg*) were also identified, suggesting that the protective effects of the composite hydrogel may involve a multifaceted regulatory network.

To bridge the transcriptomic-proteomic gap and validate the core DEGs identified by RNA-seq, we performed Western blot analysis for Casp1, Chac1 and Sesn2 at the protein level. The protein expression trends were consistent with the transcriptomic results: OGD injury significantly upregulated the protein levels of Casp1, Chac1 and Sesn2, while GK@NPs treatment markedly reversed these changes (Supplementary Fig. 13). These results support the sequencing findings regarding mitochondrial apoptosis regulation.

### 3.8 Verification of the Akt/Bad and MAPK/Rap1 signaling pathways

Given the dual findings of rapid post-translational Akt activation and sustained transcriptional MAPK/Rap1 enrichment, we further performed pathway validation using an AKTi to dissect the regulatory network and crosstalk between the two axes.

Loss of MMP is an early hallmark of apoptosis. JC-1 staining results showed that composite hydrogel incubation significantly alleviated mitochondrial dysfunction, with the drug-loaded composite hydrogel providing the strongest protection of MMP. AKTi treatment partially abrogated the protective effect of the hydrogel on mitochondrial membrane potential (Fig. 8a and b). Flow cytometry quantification further confirmed that hydrogel intervention markedly reduced apoptosis, while AKTi partially reversed the anti-apoptotic effect (Fig. 8c and d).

**Fig. 8:**
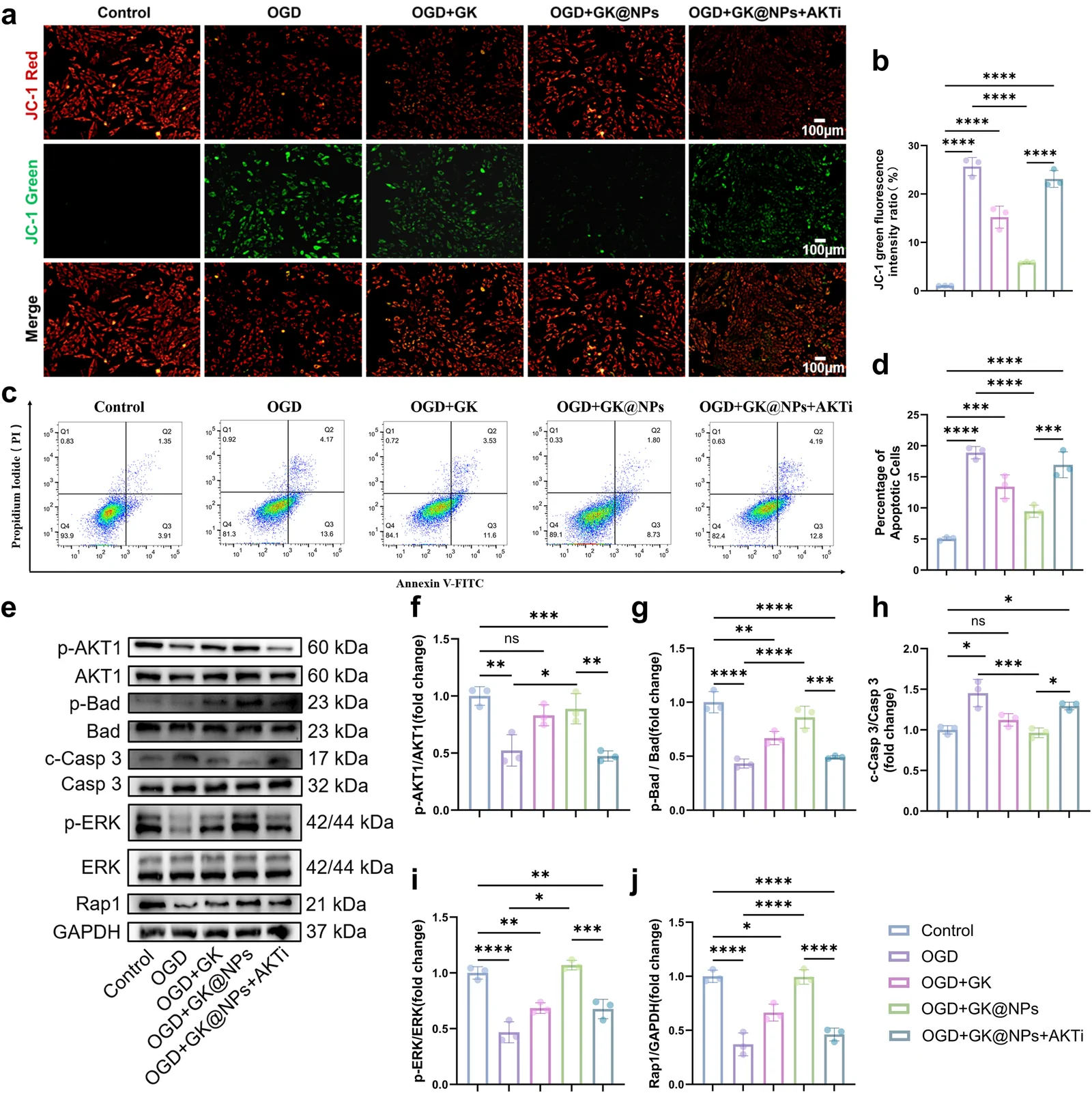
Verification of the mitochondrial apoptosis pathway via Akt inhibition in H9C2 cardiomyocytes. (a) JC-1 staining for mitochondrial membrane potential. (b) Quantitative analysis of JC-1 green fluorescence ratio. (c) Annexin V-FITC/PI flow cytometry for cell apoptosis. (d) Quantitative analysis of apoptotic cell percentage. (e) Western blot analysis of Akt/Bad, ERK and Rap1 pathway proteins with or without AKTi treatment. (f–j) Quantitative analysis of relative protein expression: (f) p-AKT1/AKT1, (g) p-Bad/Bad, (h) c-Casp3/Casp3, (i) p-ERK/ERK, (j) Rap1/GAPDH. (Data presented as mean ± SD, n = 3, ns: no significant differences, *P < 0.05, **P < 0.01, ***P < 0.001, ****P < 0.0001). (For interpretation of the references to colour in this figure legend, the reader is referred to the Web version of this article.)

Western blot analysis demonstrated that hydrogel treatment restored the phosphorylation levels of Akt1 and Bad, confirming activation of the Akt/Bad survival axis. In parallel, we validated the core effectors of the transcriptionally enriched MAPK and Rap1 pathways at the protein level. The protein levels of phosphorylated ERK (the canonical effector of the MAPK cascade) and Rap1 were significantly downregulated after OGD injury and restored by GK@NPs treatment. AKTi administration partially decreased ERK phosphorylation and Rap1 expression, suggesting molecular crosstalk between the Akt/Bad axis and the MAPK/Rap1 pathway (Fig. 8e–j). Immunofluorescence staining was performed to further validate the involvement of the Akt/Bad axis and apoptotic execution. At 4 h post-OGD, the fluorescence signals of p-AKT1 and p-Bad were significantly decreased in OGD-injured cells, which were notably restored following composite hydrogel (OGD + GK@NPs) treatment, and these restorative effects were blunted by AKTi (Fig. 9a, 9b, 9d, 9e). At 8 h post-OGD, OGD injury induced massive cell shrinkage (observed via Phalloidin staining) and a significant increase in cleaved caspase-3 (c-Casp3) fluorescence, which were effectively attenuated by the composite hydrogel treatment but reversed by AKTi (Fig. 9c and f).

**Fig. 9:**
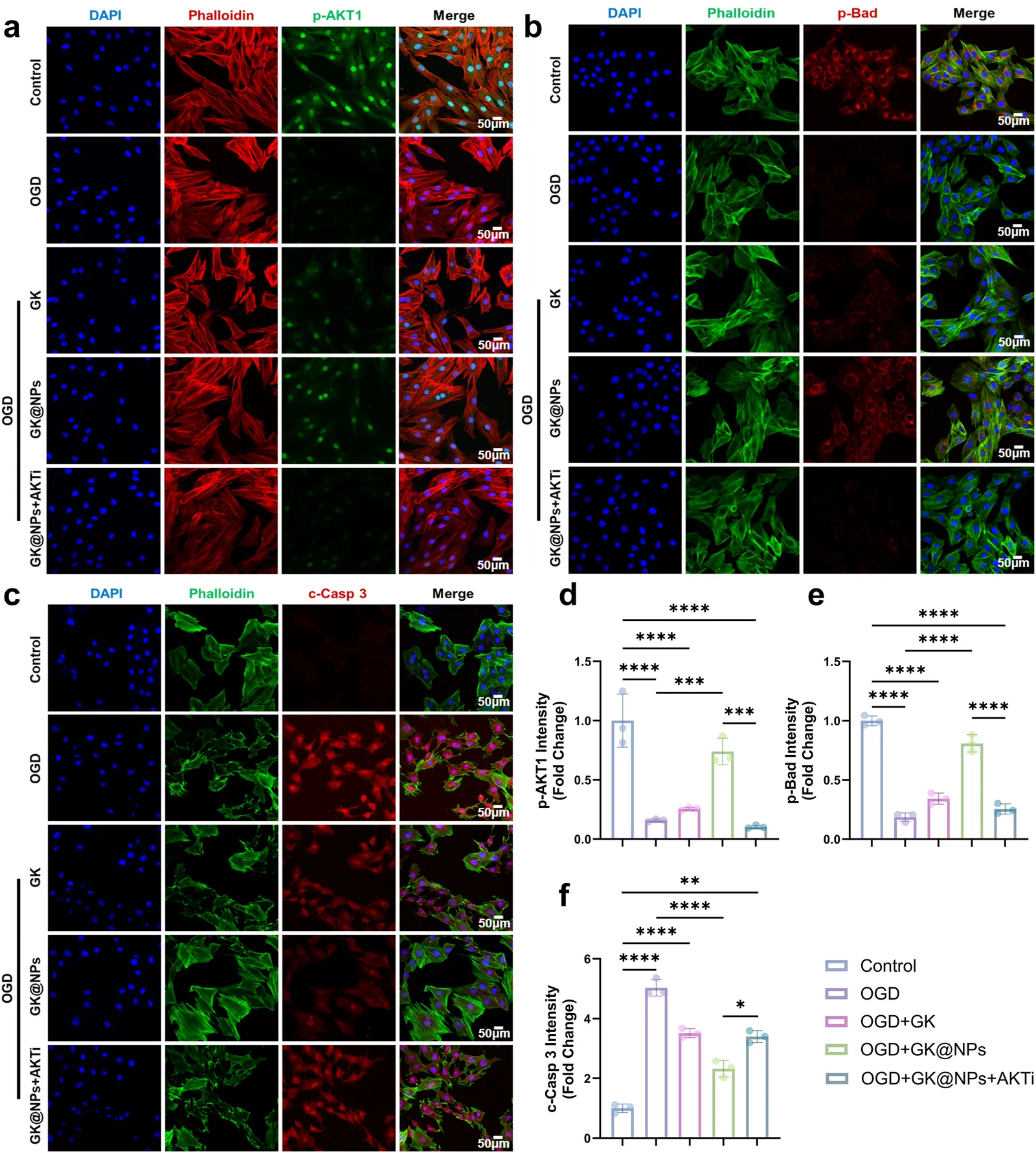
Immunofluorescence validation of the Akt/Bad-mediated mitochondrial apoptosis pathway in OGD-injured H9C2 cells. (a) Co-immunofluorescence staining of p-AKT1, phalloidin and DAPI. (b) Co-immunofluorescence staining of p-Bad, phalloidin and DAPI. (c) Co-immunofluorescence staining of c-Casp3, phalloidin and DAPI. (d–f) Quantitative fluorescence intensity analysis: (d) p-AKT1, (e) p-Bad, (f) c-Casp3. (Data presented as mean ± SD, n = 3, *P < 0.05, **P < 0.01, ***P < 0.001, ****P < 0.0001).

Collectively, the cardioprotective effect is co-mediated by two temporal layers of regulation: (1) rapid post-translational activation of the Akt/Bad axis, which acutely suppresses mitochondrial apoptosis, and (2) sustained Rap1/MAPK signaling, which drives transcriptional reprogramming of downstream apoptosis-related genes (e.g., *Casp1*, *Chac1*, *Sesn2*) to exert long-term cytoprotective effects.

### 3.9 Composite hydrogel inhibits fibroblast activation and migration and promotes endothelial angiogenesis

An in vitro fibrosis model was established by TGF-β1 stimulation. Results showed that TGF-β1 significantly upregulated α-SMA protein expression in RCFs, inducing excessive fibroblast activation. Blank GK hydrogel alone moderately reduced fibroblast activation, while the GK@NPs composite system demonstrated a significantly more pronounced inhibitory effect (Fig. 10a–d). Western blot and immunofluorescence results corroborated each other, confirming that the composite hydrogel effectively downregulated α-SMA expression, thereby suppressing the pathological transdifferentiation of cardiac fibroblasts into myofibroblasts at the source.

**Fig. 10:**
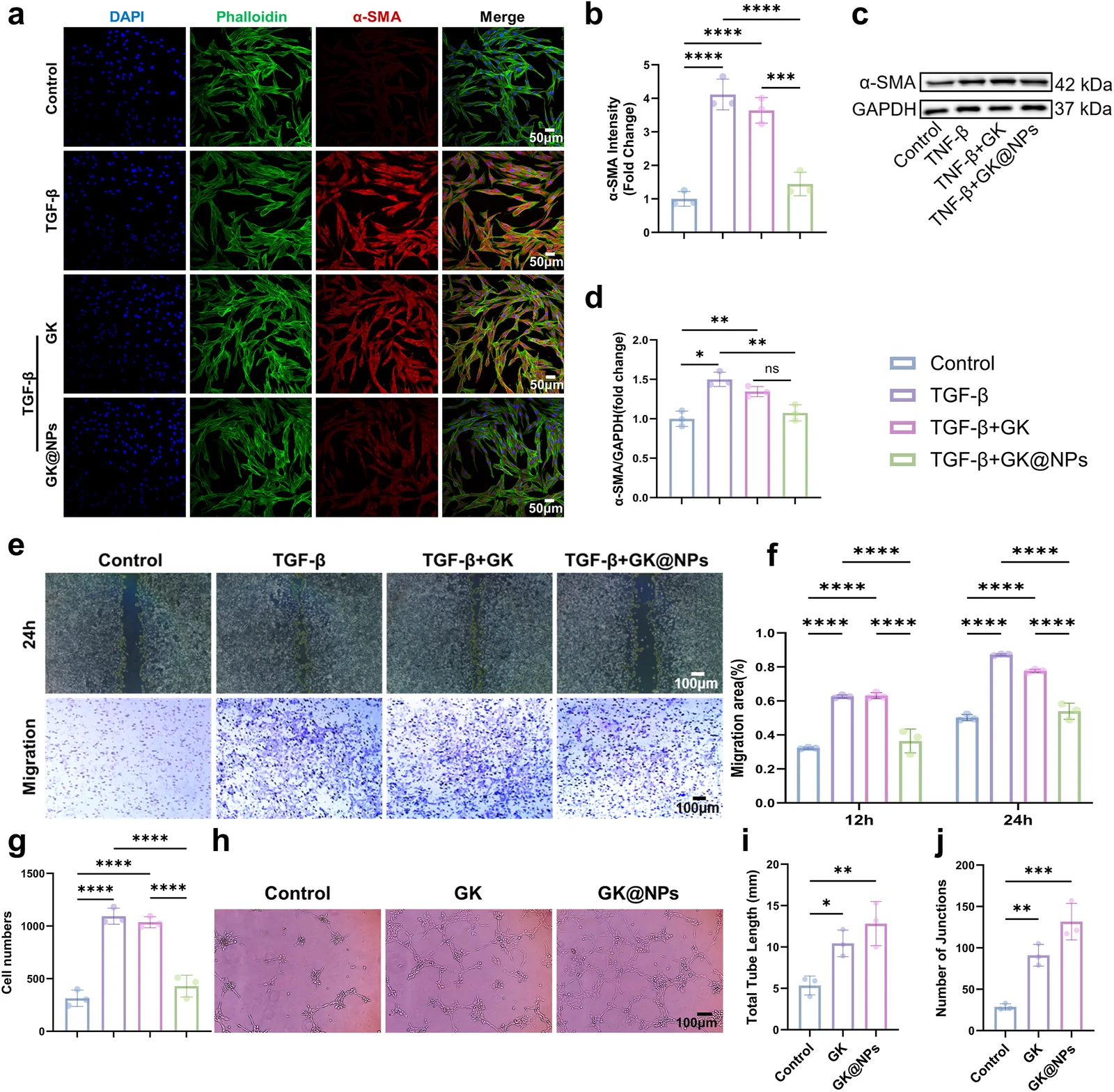
Biological effects of composite hydrogels on RCF activation, cell migration, and HUVEC tube formation. (a) Co-immunofluorescence staining of α-SMA, phalloidin and DAPI in RCFs. (b) Quantitative analysis of α-SMA fluorescence intensity. (c) Western blot of α-SMA. (d) Quantitative analysis of α-SMA protein level. (e) Scratch wound healing and Transwell migration assays. (f) Quantitative analysis of migration area ratio at 12 h and 24 h. (g) Quantitative statistics of migrated cell numbers. (h) In vitro tube formation assay of HUVECs. (i,j) Quantitative analysis of total tube length (i) and number of junctions (j). (Data presented as mean ± SD, n = 3, ns: no significant differences, *P < 0.05, **P < 0.01, ***P < 0.001, ****P < 0.0001).

Scratch wound healing and Transwell migration assays showed that TGF-β1 significantly enhanced the migratory and invasive capacity of cardiac fibroblasts, with the number of migrated cells reaching (1093 ± 76) cells/field at 24 h; following GK@NPs composite hydrogel intervention, fibroblast migration was significantly inhibited and the number of migrated cells was substantially reduced to (429.7 ± 103.9) cells/field (Fig. 10e–g).

The pro-angiogenic potential of the composite hydrogel was evaluated using an HUVEC in vitro tube formation assay (Fig. 10h–j). In the blank control group, HUVECs exhibited poor tube-forming capacity, with sparse, short tubular structures and poor network integrity; total tube length was only (5.34 ± 1.34) mm with (28.7 ± 3.8) nodes. In comparison, hydrogel-conditioned medium effectively improved endothelial tube morphology and promoted vascular network construction; the GK@NPs group demonstrated the most pronounced pro-angiogenic effect, with total tube length increasing to (12.8 ± 2.67) mm and branch nodes increasing to (131.7 ± 22.0), demonstrating favorable pro-angiogenic activity.

## 4 Discussion

Developing multifunctional biomaterials that integrate controlled drug delivery, antioxidant activity, and tissue regeneration capacity represents a promising strategy for MI treatment [33], [34]. In this study, we constructed a pH-responsive sustained-release composite hydrogel (based on GK) encapsulating luteolin-loaded PLGA nanoparticles. In this pH-responsive GK@NPs hydrogel, carboxyl protonation reversibly reduces swelling and modulus without cleaving the covalent backbone, whereas accelerated release and mass loss arise mainly from acid-catalyzed PLGA hydrolysis; in a rat MI model, the patch reduced apoptosis and collagen deposition, promoted angiogenesis, and improved systolic and diastolic function.

Wang et al. [35] reported a gelatin patch that reduced post-MI inflammation and fibrosis through M2 macrophage polarization; in contrast, moderate KerMA incorporation (0.5%) in our system tunes both mechanics and the cell-adhesion microenvironment [36]. Nevertheless, excessive KerMA content (≥1.0%) should be avoided. Higher KerMA fractions (e.g., GK-1.0 and GK-1.5) elevate photo-crosslinking density and shrink hydrogel mesh size, which partially shields RGD integrin-binding motifs and hinders focal adhesion maturation, consequently restricting spreading of H9C2 cardiomyocytes and RCFs [37]. Increased crosslinking also raises matrix stiffness beyond the optimal range for cardiac cells. Excess KerMA also raises stiffness beyond the cardiac-cell optimal range [38]. Notably, the favorable cell spreading observed in vitro reflects surface cytocompatibility under culture conditions and should not be interpreted as evidence of enhanced epicardial adhesion in vivo, where the patch is applied to the outer surface and does not directly contact cardiomyocytes. The moderate adhesion of GK-0.5 should therefore be viewed as a cytocompatibility readout rather than a design goal; excessive interfacial adhesion may instead promote clinically undesirable postoperative pericardial adhesion. Future antifouling or tunable surface-modification strategies, together with dedicated large-animal implantation studies using standardized pericardial adhesion scoring, will be required to balance stable tissue integration against adhesion-related complications [39].

pH-responsive PLGA nanoparticles were integrated into the hydrogel matrix to achieve local drug accumulation at infarcted lesions, thereby reducing off-target effects associated with systemic administration. Unlike the sustained delivery approaches adopted by Taiwaikuli et al. [40] and Wang et al. [41], our pH-triggered platform is better adapted to the acidic infarct microenvironment. The scaffold shows modest reversible softening at pH 6.5, improving compliance with cardiac pulsation and reducing mechanical compression on fragile infarcted tissue. The accelerated release under acid is attributable mainly to PLGA hydrolysis rather than to loosened network crosslinks; despite the modulus change, the covalent network retained structural integrity and provided mechanical support to the infarcted wall, consistent with previous pH-responsive cardiac hydrogel systems [18].

Luteolin is a flavonoid with multitarget biological activity. Our study investigated its mechanism in MI, particularly Akt/Bad signaling activation to inhibit mitochondrial apoptosis. This is consistent with the work of Li et al. [29], who reported the role of PI3K-Akt/HIF-1α in myocardial fibrosis, and extends our understanding of Akt downstream effectors. In the OGD model, we observed a temporal discrepancy between apoptotic signal transduction and morphological changes in H9C2 cells. Downregulation of p-Bad/p-AKT1 occurred at the early stage of OGD treatment (4 h), when cellular structure remained largely intact, whereas pronounced nuclear morphological changes and nuclear translocation of cleaved caspase-3 appeared at later time points. This finding is consistent with the cascade characteristics of the intrinsic apoptosis pathway [42], [43], [44]. The early time window appears critical for reversing OGD-induced suppression of p-AKT1/p-Bad signaling. This blocks the apoptotic program at its upstream and may reduce late-stage cellular damage. These findings are attributable to the synergistic effect of the multitarget properties of luteolin and the protective microenvironment provided by the hydrogel scaffold.

Transcriptomic data revealed that the composite material modulated a broader molecular network, including the MAPK and Rap1 signaling pathways—both well-established regulators of cell survival and stress responses [45], [46], [47]. A divergence was observed between the transcriptomic and proteomic results. This is consistent with the established paradigm that small-molecule drugs can activate pro-survival pathways such as Akt within minutes to hours [48], [49]. Mechanistically, rapid Akt phosphorylation acts as an early signal to halt the mitochondrial apoptotic cascade. In contrast, Rap1/MAPK signaling drives longer-term transcriptional adaptation of cellular stress responses [50]. The two axes are not linearly separated: Akt and MAPK pathways are known to engage in bidirectional crosstalk via shared upstream nodes and feedback loops. In our AKTi rescue experiments, GK@NPs-induced upregulation of p-ERK and Rap1 was partially attenuated, indicating functional interplay between the Akt/Bad and Rap1–MAPK axes rather than strict hierarchical dependence [51].

The pathological process following MI is not unitary but rather a cascading response involving multiple pathological links [52], [53]. The patch targeted three injury axes. (i) Anti-apoptotic: GK@NPs reversed OGD-induced cardiomyocyte apoptosis through luteolin and the biomimetic porous scaffold [24], [54], [55]. (ii) Anti-fibrotic: pathological fibrosis after MI drives progressive cardiac deterioration [56], [57]; TGF-β drives myofibroblast conversion and ECM deposition [58], [59], and we observed reduced TGF-β only in the acute-to-subacute phase, representing finite local release rather than chronic blockade, since unrestrained TGF-β suppression may impair scar maturation and risk wall thinning or rupture [60], [61], [62]. (iii) Pro-angiogenic: VEGF upregulation arose from luteolin activity, scaffold guidance, and indirect TGF-β modulation.

Regarding in vivo residence, DiR fluorescence tracking of the encapsulated nanoparticles showed sustained epicardial signal through day 14 and a marked decline by day 28 (Supplementary Fig. 7), consistent with previous epicardial GelMA bioadhesives [63], and with the post-infarction remodeling window. Because DiR reports carrier fluorescence rather than patch mass, this serves as an indirect proxy for residence; direct in vivo gravimetric degradation kinetics were not measured in this study.

Several limitations apply. Although in vivo blank-matrix and blank-carrier controls (MI + GK, MI + GK@PLGA) were included, free-luteolin, hydrogel-free bare-NP, and in vitro GK@PLGA arms were not performed, and the pathway was validated only in H9C2 cells without in vivo tissue Western blot or transcriptomics; long-term outcomes beyond 28 days were also not assessed. Direct in vivo patch degradation and local intramyocardial pH were not measured—DiR fluorescence serves only as a residence proxy and pH 6.5 was a literature-based in vitro condition—leaving these controls, tissue-level mechanism, gravimetric degradation, and spatiotemporal pH mapping for future work.

In summary, the GK@NPs composite hydrogel achieves cardiac protection through the synergistic interplay of structural reinforcement and multi-signal regulation. Nevertheless, the current system does not fully recapitulate the evolving pathological stages of MI, and the spatiotemporal precision of drug release could be further improved. Programmable delivery platforms that sense post-infarction microenvironmental cues and exhibit multistage responsiveness represent an ideal direction for temporally sequenced drug release.

## 5 Conclusions

In this study, a photo-crosslinked/hydrogen-bond dual-network GK@NPs hydrogel was constructed. It matches myocardial mechanics, responds to the acidic MI lesion for sustained release, scavenges ROS, reduces apoptosis and fibrosis, and promotes angiogenesis. Protection relies on coordinated rapid Akt/Bad activation and sustained MAPK/Rap1 modulation, offering a minimally invasive local-delivery approach for MI.

## CRediT authorship contribution statement

**Dongbin Jiang:** Writing – review & editing, Writing – original draft, Validation, Project administration, Methodology, Formal analysis, Data curation. **Jiaying Li:** Writing – review & editing, Writing – original draft, Supervision. **Junkai Hu:** Writing – review & editing, Writing – original draft, Supervision. **Dan Jiang:** Writing – review & editing, Resources. **Weihu Yang:** Writing – review & editing, Supervision, Resources. **Wenhua Huang:** Writing – review & editing, Resources, Supervision.

## Ethics approval and consent to participate

All animal experiments were performed in accordance with the Guide for the Care and Use of Laboratory Animals, and this study was approved by the Ethics Committee of Southern Medical University, Guangzhou, China.

## Publication consent

Publication consent has been obtained from the participants.

## Funding

This research was supported by the 10.13039/501100012166National Key R&D Program of China (2022YFB4600600).

## Declaration of competing interest

The authors declare that they have no known competing financial interests or personal relationships that could have appeared to influence the work reported in this paper.

## Data Availability

Data will be made available on request.
