# Supplementary material for: A pH-responsive GelMA-KerMA hydrogel delivering luteolin-loaded PLGA nanoparticles promotes myocardial repair via Akt/Bad signaling and multitarget regulation
Source: Mater Today Bio. 2026 Oct 31;41:103791. doi: 10.1016/j.mtbio.2026.103791 (PMC13756189; doi:10.1016/j.mtbio.2026.103791)
Supplement: Supplementary file 1 [file mmc1.docx]

Supporting Information

**A pH-Responsive GelMA-KerMA Hydrogel Delivering Luteolin-Loaded PLGA Nanoparticles Promotes Myocardial Repair via Akt/Bad Signaling and Multitarget Regulation**

Dongbin Jiang¹, Jiaying Li², Junkai Hu¹, Dan Jiang², Weihu Yang³*, Wenhua Huang¹,²**

Affiliations:

¹ Department of Surgery, The First School of Clinical Medicine, Guangdong Medical University, Zhanjiang, China

2 Guangdong Engineering Research Center for Translation of Medical 3D Printing Application, Guangdong Provincial Key Laboratory of Digital Medicine and Biomechanics, National Key Discipline of Human Anatomy, School of Basic Medical Sciences, Southern Medical University, Guangzhou, China.

3 Key Laboratory of Biorheological Science and Technology, Ministry of Education College of Bioengineering, Chongqing University, Chongqing, China.

* Corresponding author. Ministry of Education College of Bioengineering, Chongqing University, 400044, Chongqing, China.

** Corresponding author. School of Basic Medical Sciences, Southern Medical University, 510515, Guangzhou, China.

E-mail addresses: yangweihu@cqu.edu.cn (W. Yang), huangwenhua2009@139.com (W. Huang).

Supplementary Fig. 1 (a) UV-vis absorption spectrum of luteolin showing the maximum absorption wavelength (λmax) at 355 nm. (b) Standard calibration curve of luteolin at 355 nm.

Supplementary Table 1 Encapsulation efficiency and drug loading of luteolin-loaded PLGA nanoparticles (n = 3)

| **Parameter** | **Value(Mean ± SD,%)** |
| --- | --- |
| Encapsulation efficiency (EE) | 84.1 ± 2.6 |
| Drug loading (DL) | 5.35 ± 0.28 |

Abbreviations: EE, encapsulation efficiency; DL, drug loading.

Supplementary Fig. 2 XPS survey spectra of hydrogel samples showing characteristic peaks of C 1s, N 1s, O 1s, and S 2p.

Supplementary Fig. 3 Amplitude sweep (a) and frequency sweep (b) rheological curves under pH 6.5 and pH 7.4 conditions.

Supplementary Fig. 4 In vitro drug release behavior of bare Lut-PLGA nanoparticles at pH 7.4 and pH 6.5. (Data presented as mean ± SD, n = 3. *****P* < 0.0001 by two-way ANOVA).

Supplementary Fig. 5 Hemolysis ratio measurements of different materials. (Data presented as mean ± SD, n = 3, ns: no significant differences).

Supplementary Fig. 6 Echocardiographic assessment of cardiac function in rats on Day 1 after myocardial infarction (MI). (a) Representative M-mode echocardiograms of rats in the MI, MI+GK, MI+GK@PLGA, and MI+GK@NPs groups. (b, c) Quantitative analysis of left ventricular fractional shortening (LVFS) and left ventricular ejection fraction (LVEF) across the four groups. (Data presented as mean ± SD, n = 6, ns: no significant differences).

Supplementary Fig. 7 In vivo tracking and transmural distribution of DiR-labeled GK@NPs loaded hydrogel patch after epicardial implantation in MI rats. (a) Representative in vivo IVIS fluorescence images of rats at day 1, 3, 7, 14 and 28 post-operation. (b) Quantitative analysis of residual DiR fluorescence signal (%) derived from IVIS imaging. (c) Quantification of DiR mean fluorescence intensity in three transmural myocardial regions (epicardium, mid-myocardium, ischemic core) from frozen heart sections at day 1, 3, 7 and 14. (d) Representative immunofluorescence merged images of full-thickness myocardial frozen slices showing DiR distribution at indicated time points. (Data presented as mean ± SD, n = 6, ns: no significant differences, **P* < 0.05, *****P* < 0.0001).

Supplementary Fig. 8 Representative immunofluorescence images of myocardial tissue sections. (a) Representative TUNEL (green), cTnT (red), DAPI (blue) and merged fluorescence images of heart sections from Sham, MI, MI+GK, MI+GK@PLGA and MI+GK@NPs groups. (b) Representative immunofluorescence images showing CD31 (green), α-SMA (red), DAPI (blue) and merged channels in each group.

Supplementary Fig. 9 (a) In vivo biosafety evaluation of composite hydrogels. H&E staining of major organs (lung, liver, spleen, kidney) from different groups at postoperative day 28. (b, c) ELISA quantification of TGF-β (b) and VEGF (c) levels in myocardial tissue. (Data presented as mean ± SD, n = 3, **P* < 0.05, ***P* < 0.01, ****P* < 0.001, *****P* < 0.0001).

Supplementary Fig. 10 Original membrane scans corresponding to the cropped bands shown in Fig. 6k. Blots for p-AKT1, AKT1, p-Bad, Bad, Bax, Bcl-2, c-Casp 3, Casp 3, cytochrome c (Cyto Cyt-c) and GAPDH are shown. Red boxes indicate the regions cropped and displayed in Fig. 6k. Molecular weight markers are shown beside each membrane.

Supplementary Fig. 11 Western blot quantification of mitochondrial apoptosis-related proteins and original membrane scans. (a–c) Quantitative analysis of relative protein expression levels of Bcl-2, Bax and cytoplasmic cytochrome c (Cyto Cyt-c), normalized to GAPDH. (d) Representative cropped bands of mitochondrial cytochrome c (Mito Cyt-c) and COX IV. (e, f) Original membrane scans for Mito Cyt-c and COX IV; red boxes indicate the regions cropped for panel d. (g) Quantitative analysis of mitochondrial cytochrome c expression normalized to COX IV. (Data presented as mean ± SD, n = 3, **P* < 0.05, ***P* < 0.01, ****P* < 0.001, *****P* < 0.0001).

Supplementary Fig. 12 Supplementary transcriptomic sequencing results from H9C2 cells. (a) Hierarchical clustering heatmap of DEGs between the OGD group and the OGD + GK@NPs group. (b) KEGG pathway enrichment bubble plot showing top-ranked enriched signaling pathways; bubble size represents gene count and color represents P value. (c) Statistical distribution of GO biological process terms.

Supplementary Fig. 13 (a) Representative cropped Western blot bands of Casp1, Chac1, Sesn2 and GAPDH from Control, OGD and OGD+GK@NPs groups. (b–e) Original scanned membrane regions with molecular weight markers, corresponding to the cropped bands shown in panel (a). Red boxes indicate the areas used for the cropped images. (f–h) Quantitative analysis of relative protein expression levels (normalized to GAPDH). (Data presented as mean ± SD, n = 3, ns: no significant differences, **P* < 0.05, ***P* < 0.01, ****P* < 0.001, *****P* < 0.0001).

Supplementary Fig.14 Original scanned membrane regions corresponding to the cropped bands shown in Figure 8e. Blots for p-AKT1, AKT1, p-Bad, Bad, c-Casp 3, Casp 3, p-ERK, ERK, Rap1 and GAPDH are shown. Red boxes indicate the regions cropped and presented in Figure 8e. Molecular weight markers are displayed beside each membrane.

Supplementary Fig. 15 Original scanned membrane regions corresponding to the cropped bands shown in Figure 10c. Blots for α-SMA and GAPDH are shown. Red boxes indicate the regions cropped and presented in Figure 10c. Molecular weight markers are displayed beside each membrane.
